# Predator avoidance promotes interbacterial symbiosis with myxobacteria in polymicrobial communities

**DOI:** 10.1093/ismejo/wrag140

**Published:** 2026-06-01

**Authors:** Shailaja Khanal Pokharel, Sheila Walsh, Nawal Shehata, Andrew Ahearne, Daniel Belin, Britney Larson, Benjamin Tabor, Daniel Wall, David Cole Stevens

**Affiliations:** Department of Biomolecular Sciences, School of Pharmacy, University of Mississippi, Oxford, MS 38677, United States; Department of Molecular Biology, University of Wyoming, 1000 E University Avenue, Laramie, WY 82071, United States; Department of Biomolecular Sciences, School of Pharmacy, University of Mississippi, Oxford, MS 38677, United States; Department of Biomolecular Sciences, School of Pharmacy, University of Mississippi, Oxford, MS 38677, United States; Department of Molecular Biology, University of Wyoming, 1000 E University Avenue, Laramie, WY 82071, United States; Department of Molecular Biology, University of Wyoming, 1000 E University Avenue, Laramie, WY 82071, United States; Department of Biomolecular Sciences, School of Pharmacy, University of Mississippi, Oxford, MS 38677, United States; Department of Molecular Biology, University of Wyoming, 1000 E University Avenue, Laramie, WY 82071, United States; Department of Biomolecular Sciences, School of Pharmacy, University of Mississippi, Oxford, MS 38677, United States

**Keywords:** myxobacteria, *Archangium*, *Microvirga*, bacterial symbiosis, predation avoidance, horizontal gene transfer, soil microbiome

## Abstract

Myxobacteria are predatory soil bacteria with the largest known bacterial genomes, rich in biosynthetic gene clusters for specialized metabolites. Despite their ecological importance as potential keystone taxa in soil food webs, there is a disconnect between laboratory-isolated myxobacteria and abundant *Myxococcota* detected in environmental metagenomic studies. Here, we report the isolation and characterization of stable myxobacterial swarm consortia from rhizospheric soil, consisting of myxobacteria associated with novel *Microvirga* species. Using metagenomic sequencing, we assembled metagenome-assembled genomes for four consortia, revealing phylogenetically distinct yet stably associated bacterial partnerships. Comparative genomics identified evidence of horizontal gene transfer, including acyl-homoserine lactone synthases and ankyrin repeat (ANKYR) proteins shared between consortium members, and genome-scale metabolic modeling predicted complementary auxotrophies. Time-lapse microscopy revealed that *Archangium* exhibited reduced predation toward its *Microvirga* companion (0.7% predation rate) compared to nonsymbiotic *Myxococcus xanthus* (14.9% predation rate) but maintained robust predatory capacity against *Escherichia coli* prey. These findings indicate that predation avoidance and metabolic complementarity can drive stable interbacterial symbiosis in predatory myxobacterial communities, providing foundational insights into previously overlooked myxobacterial partnerships that may be prevalent in natural soil ecosystems.

## Introduction

Members of the phylum *Myxococcota*, colloquially referred to as myxobacteria, demonstrate traits atypical of bacteria such as “wolf-pack” predatory swarming to acquire macromolecular nutrients from lysed prey and contact-dependent recognition of both kin and prey [1–5]. Nearly all myxobacteria from the class Myxococcia are considered generalist predators with prey including bacteria, fungi, and oomycetes, and their predatory capacity appears to be shaped more by environmental conditions and microbial community composition than phylogeny [6–14]. Consistent with ecological impact, recent evidence of a nutrient cycling process independent of eukaryotic micropredators highlighted myxobacteria as potential keystone taxa in the soil food web [15]. At the genomic scale, myxobacteria further distinguish themselves by maintaining the largest known bacterial genomes, rich in biosynthetic gene clusters (BGCs) that encode specialized metabolites [16–19]. Deemed “gifted” for their potential to produce biologically active metabolites, myxobacteria are targeted for genome mining for discovery of natural products which have expanded efforts to isolate novel myxobacteria from the environment resulting in the discovery of over 40 new species of myxobacteria [20–22]. These discoveries include representatives from eleven newly described genera within the last decade. However, the vast majority of laboratory and type strain myxobacteria are minimally present in environmental metagenomic data, and there is a disconnect between myxobacteria with sequenced genomes and abundant *Myxococcota* from ecological, metagenomic analysis of soil [23–25].

The observation that myxobacteria select for diverse prey during predation may provide insight into this discrepancy with metagenomic sampling [26]. Results from controlled predator–prey experiments have repeatedly demonstrated selection of prey phenotypes that influence mucoidy, metabolism, cofactor access, and growth [27–31]. Variable prey ranges of myxobacterial isolates suggest these trophic interactions likely scale to environmental conditions in soil and influence microbial community structure [8, 24]. We hypothesize that predator–prey coevolution may result in symbiotic relationships between myxobacteria and prey that avoid predation. Predation-resistant neighbors in polymicrobial communities could benefit from shared goods released during myxobacterial lysis of prey populations susceptible to predation. Although laboratory predator–prey experiments have resulted in predation-resistant prey, the rapid response and phenotypes observed could also be attributed to general stress responses [28, 32–35]. Unfortunately, time and resource constraints limit the likelihood of observing selection of prey resistance and subsequent symbiosis with controlled predator–prey experiments. Similarly, metagenomic investigation of natural polymicrobial communities have provided corollary evidence of associations between myxobacteria and nonmyxobacteria, but these analyses cannot elucidate specific symbiotic relationships involving myxobacteria [13, 14, 25]. Ultimately, myxobacteria-inclusive consortia isolated from the environment, which are stable and amenable to repeated experimental conditions, are the ideal model to study microbial community structure and possible symbiosis.

Twenty-nine years ago, five isolates of the myxobacterium *Chondromyces crocatus* were reported to be associated with a “companion” bacterium, *Candidatus comitans* [36, 37]. Inferring from 16S rRNA gene sequence homology and observed production of sphingolipids, *Ca. comitans* was determined to be closely related to members of the genus *Sphingobacterium* and was unable to survive passages as a monoculture removed from co-culture conditions with *Ch. crocatus*. These myxobacteria–companion pairings would be ideal models for comparative genomic analyses and subsequent, controlled experiments to explore symbiotic traits. However, their discovery predated routine genomics, and no further instances of naturally occurring myxobacterial consortia have been reported. During our efforts to isolate myxobacteria from soil, we occasionally encounter isolates that we cannot obtain as monocultures, despite repeated passages, and we have also previously reported a contaminant or perhaps companion *Aneurinibacillus* sp. capable of surviving co-culture conditions with the myxobacterium *Archangium violaceum* [38]. Searching for myxobacteria-inclusive consortia, similar to the discoveries of Jacobi *et al*., we sequenced four xenic isolates from rhizospheric soil that were obtained using prey-baiting methodology, but these isolates were recalcitrant to yielding myxobacterial monocultures. Mirroring observations from Jacobi *et al*., these natural swarm consortia grow on solid media as xenic, circular swarms. Herein, we report the resulting identities of consortia members, comparative metagenomic findings, and show a companion is specifically resistant to predation by a myxobacteria swarm consortium.

## Materials and Methods

### Isolation and cultivation of swarm consortia

All swarm consortia were isolated from rhizospheric soils samples using methodology previously described for isolating myxobacteria from soil [20]. Briefly, a lawn of *Escherichia coli* was cultivated overnight at 37°C on Luria–Bertani (LB) agar (1.5%). The resulting biomass was scraped into 2 ml of antifungal solution containing 250 μg/ml cycloheximide and nystatin. Approximately 300 μl of this suspension was then spread onto the center of Water agar (WAT) plates to generate a bait circle roughly 2 inches in diameter. Plates were allowed to air-dry to form *E. coli*–WAT bait plates. Separately, previously air-dried soil was rehydrated with the same antifungal solution to a mud-like consistency. Once the *E. coli* lawn on the WAT plate had dried, a pea-sized portion of the soil paste was placed at the center of the bait circle. Inoculated plates were incubated at 25°C for up to 4 weeks and monitored daily for the development of lytic zones or fruiting bodies within the *E. coli* lawn. Emerging lytic zones were transferred using a sterile syringe needle onto VY/4 agar (2.5 g/L Baker’s yeast, 1.36 g/L CaCl_2_·2H_2_O, 0.5 mg/L vitamin B12, 15 g/L agar). The advancing swarm edge was repeatedly subcultured onto fresh VY/4 plates to obtain isolated colonies. However, throughout this purification process, the four samples, WIMLSP1, WIMLSP2, DLMAZ, and FLWO, consistently formed xenic swarms and remained resistant to monoculture despite multiple rounds of passaging. All swarm consortia were maintained on VY/4 plates and liquid cultures with CYH/2 media (0.75 g/L of casitone, 0.75 g/L of yeast extract, 2 g/L of starch, 0.5 g/L of soy flour, 0.5 g/L of glucose, 0.5 g/L of MgSO_4_•7H_2_O, 1 g/L of CaCl_2_•2H_2_O, 6 g/L of HEPES, 8 mg/L of EDTA-Fe, and 0.5 mg/L of vitamin B_12_). WIMLSP1 and WIMLSP2 were isolated from soil collected in Spring 2021 from the roots of a white spruce tree near White Lake, MI, USA (42.35, −84.35). FLWO was isolated from soil collected in Spring 2022 from the roots of a white oak tree near Palm Coast, FL, USA (29.53, −81.22). DLMAZ was isolated from soil collected in Spring 2021 from the roots of a New Mexico desert locust tree near Mesa, Arizona, USA (33.41, −111.83).

### Scanning electron microscopy

Bacterial samples intended for scanning electron microscopy (SEM) were prepared using a standard fixation-dehydration protocol optimized for microbial colonies [39]. Briefly, cells from each swarm consortium or monoculture myxobacterium after 72 h of growth on VY/4 agar were fixed in 2.5% glutaraldehyde prepared in 0.1 M phosphate buffer (pH 7.2) for 45 min–1 h, followed by 15-min wash in the same buffer. Samples were then re-fixed in 1% osmium tetroxide in phosphate-buffered saline (PBS) buffer for 60 min at room temperature to enhance membrane contrast and subsequently rinsed with buffer. Fixed samples were dehydrated through a graded ethanol series (30%, 50%, 70%, 90%, 95%, and 100%, in 10 min each), and the samples were dried overnight in a flow hood. Dehydrated samples were mounted onto aluminum stubs using carbon adhesive tape. Mounted samples were sputter-coated with a 10–20 nm layer of gold–palladium alloy using a Denton Vacuum Desk V TSC Sputter Coater and subsequently examined using a JEOL JSM-7200FLV Field-Emission Scanning Electron Microscope in the Glycore Imaging and Microscopy Core for SEM analysis.

### Metagenomic sequencing

High–molecular-weight metagenomic DNA was extracted from xenic swarm consortia using Qiagen Genomic-Tip columns. DNA concentration and purity were assessed using Qubit dsDNA HS Assay Kits (ThermoFisher Scientific) and a Nanodrop spectrophotometer. Sequencing libraries were prepared according to the manufacturer’s instructions for the Native Barcoding Kit 24 V14 (Oxford Nanopore). The barcoded library was then loaded onto an Oxford Nanopore MinION flow cell (R10.4.1) and sequenced until ~1–2 Gbases of data per barcode were obtained. Raw nanopore reads were basecalled and demultiplexed with Dorado (v0.7.2) using the super-accurate model. Metagenome assembly was carried out with Flye (v2.9.2+), and consensus polishing was performed using Medaka (v1.11+) [40, 41]. Resulting assemblies were deposited at the Joint Genome Institute Integrated Microbial Genomes & Microbiomes (JGI IMG/MER) database. Statistics and consortia details from assemblies are provided as Supplemental Tables (Tables S1–S5).

### Comparative genomic analysis

Annotation of sequenced swarm consortium metagenomes was completed using the JGI IMG annotation pipeline (v5.2.1) [42]. Individual metagenome-assembled genomes (MAGs) from each swarm consortium were analyzed at the Type Strain Genome Server (TYGS) to acquire digital DNA–DNA hybridization (dDDH) and 16S rRNA gene sequence comparisons [43–45]. Phylogenetic trees generated at the TYGS (v409) and dDDH values are included as Supplemental data (Tables S6 and S7, Figs S1–S4). Average nucleotide identity values were calculated using the OrthoANI Tool (OAT) (v0.93.1) [46]. Genome-scale metabolic models were constructed and characterized from MAGs using the MS2 Build metabolic models with OMEGGA (v2.0) and Run Model Characterization (v2.2.1) applications in KBase [47–49]. Amino acid auxotrophies for each MAG were also calculated using GapMind [50]. KEGG modules, KEGG pathways, and gene phylogenies associated with each metagenome were provided as part of the JGI IMG annotation pipeline and were accessed from the JGI IMG/MER database [42, 51–53]. MEGA X (v10.1.7) was used for alignments of acyl-homoserine lactone (AHL) synthases and ANKYR proteins and construction of phylogenetic trees [54]. Predicted structures for AHL synthases and ANKYR proteins were modeled using the AlphaFold Server (AlphaFold 3) and subsequently aligned using the FoldMason Multiple Protein Structure Alignment tool (FoldMason MSA) [55, 56]. ANKYR protein sequences from WIMLSP1 and WIMLSP2 were analyzed using the Enzyme Function Initiative Enzyme Functionality Tool (ESI-EST) to generate sequence similarity networks and determine phylogenetic distribution of homologs [57–59] (Fig. S5). ANKYR protein sequences from WIMLSP1 and WIMLSP2 were analyzed using SignalP (v6.0) to determine the presence of any signal peptides. Sequence similarity networks generated by ESI-EST were subsequently utlized as inputs for Enzyme Function Initiative Genome Neighborhood Tool (EFI-GNT) to determine spatial organization of genes ANKYR encoding genes [57–59].

### Predation assays

WIMLSP2 was cultured in CY/H medium at 33°C with shaking at 285 rpm to late exponential phase to ensure each member was represented sufficiently. *Myxococcus xanthus* DK1622-GFP was grown in CTT broth (1% Casitone, 10 mM Tris–HCl pH 7.6, 8 mM MgSO₄, 1 mM KH₂PO₄) at 33°C with shaking at 300 rpm to exponential phase. *Escherichia coli* DH5α-mCherry was grown in LB supplemented with kanamycin (50 μg ml^−1^) at 37°C with shaking at 250 rpm to an OD_600_ of ~0.5.

For microscopy assays, cultures were back-diluted to ~OD_600_ 0.25 for the WIMLSP2 consortium and DK1622-GFP or to OD_600_ 0.05 for *E. coli*. Three conditions were prepared for imaging: the WIMLSP2 consortium alone, WIMLSP2 mixed with DK1622-GFP, and WIMLSP2 mixed with *E. coli*–mCherry. Mixed samples were combined at a 1:1 (vol/vol) ratio immediately before imaging. A 5 μl aliquot of each culture or culture mixture was spotted onto starvation buffer (TPM) agar pads (10 mM Tris–HCl pH 7.6, 8 mM MgSO₄, 1 mM KH₂PO₄, 1% agar) supplemented with 2 mM CaCl₂. Pads were cast onto glass microscope slides and allowed to air dry before imaging.

Time-lapse fluorescence microscopy was performed using an Olympus IX83 inverted microscope equipped with a 60× oil-immersion objective and controlled with cellSens software. GFP and mCherry fluorescence were visualized using standard fluorescein isothiocyanate (FITC) and mCherry filter sets, respectively. Images were acquired every 20 s for a total duration of 2 h. Predator–prey behaviors were scored manually from time-lapse movies. Examples of time-lapse movies depicting predator–prey behaviors are provided as Supplemental Movies (Movies S1–S4). An interaction event was defined as the first frame in which a predator cell made contact with a prey cell. If the contacted prey cell underwent visible lysis within 10 min, the event was scored as a kill; if no lysis occurred, it was scored as a nonlethal interaction. Repeated contacts between the same predator and prey cell were counted as separate interactions only when the cells were clearly separated and then re-established contact.

## Results

### Swarm consortia isolated from rhizospheric soil are stable and amenable to laboratory conditions

We sought to isolate swarm consortia using a standard prey-baiting approach that we have previously employed to isolate myxobacteria from rhizospheric soil [20, 60]. Our isolation process involves nutrient-free, minimal medium supplemented with live *E. coli* as bait; prey-baiting media are inoculated with soil and monitored for the appearance of visible swarms indicative of myxobacterial growth. Observed swarms were assumed to lyse and obtain nutrition from *E. coli* cells. Serial passages of swarms with prey-based minimal media typically yield monocultures of myxobacterial isolates. During this process, three samples WIMLSP1, FLWO, and DLMAZ produced xenic swarms that were recalcitrant to monoculture after repeat passages. Despite numerous passages, we were unable to obtain myxobacterial monocultures from WIMLSP1, FLWO, and DLMAZ consortia.

**Figure 1:**
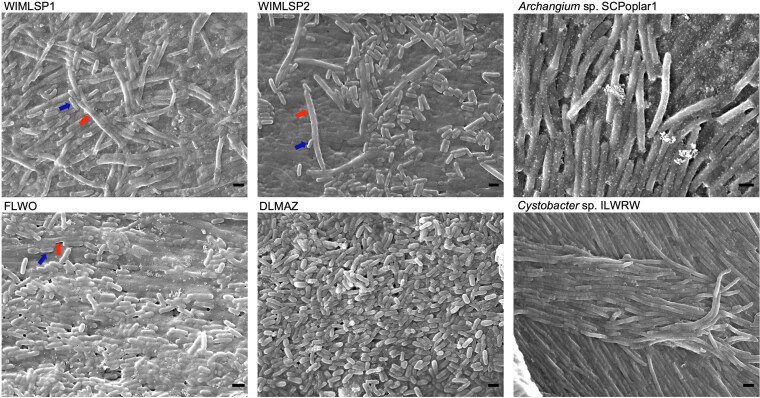
SEM images from swarm consortia WIMLSP1, WIMLSP2, FLWO, and DLMAZ and monoculture representatives *Archangium* sp. SCPoplar1 and *Cystobacter* sp. ILWRW for comparison. Representative myxobacterial (longer length) and microvirgal (shorter length) cells in swarm consortia are labelled with arrows, respectively. All scale bars correspond to 1 μm.

We attempted to reisolate WIMLSP1 from its source soil to determine the stability of the candidate swarm consortium and to help rule out the possibility we serendipitously isolated two microbes with similar cultivation conditions. Using the same isolation conditions for WIMLSP1, we were able to isolate a fourth xenic swarm consortium, WIMLSP2. SEM of swarm consortia revealed the presence of cells with two distinct sizes in WIMLSP1 and WIMLSP2 and, to a lesser extent, FLWO (Fig. 1). Larger cells with sizes ranging from 7 to 9 μM were assumed to be myxobacteria, and smaller cells (1–1.5 μM) were considered candidate companion bacteria. No larger myxobacterial cells were apparent in SEM images of DLMAZ. Subsequent cultivation revealed all four swarm consortia grew on solid and liquid media used for culturing myxobacteria (CTT, CTTYE, CY/H, and VY/2) and endured maintenance as freezer stocks.

### Metagenomic analysis reveals association between myxobacteria and *Microvirga* spp.

Using metagenomic sequencing, we sought to identify members of each swarm consortium. High-molecular-weight (HMW) metagenomic DNA was prepared from each consortium and sequenced with long-read nanopore sequencing. Two MAGs were present in each sequenced swarm consortium (Table 1). Consensus data from 16S rRNA gene comparison, dDDH, and average nucleotide identity (ANI) analysis indicated the presence of one myxobacterium and one *Microvirga* spp. in all four consortia. Coverage differences between MAGs from each metagenome assembly were noted as potential differences in abundances between consortia members at the time of HMW DNA isolation. Although fold coverage values for MAGs are not direct measurements of abundance, the differences between consortia are notable with a coverage ratio of 3.75:1 *Archangium* to *Microvirga* in WIMLSP1 and WIMLSP2 and coverage ratios of 5.5:1 and 4.2:1 *Microvirga* to *Archangium/Cystobacter* in FLWO and DLMAZ, respectively. Higher abundances of *Microvirga* in FLWO and DLMAZ may explain the relative difficulty we experienced trying to capture images of myxobacterial cells from these consortia using SEM.

**Table 1:** MAG assembly and taxonomy data for sequenced consortia strains.

	Size (Mb)	Coverage (×)	Contigs	Coding sequences	GC content (%)	16S rRNA (%)	ANI (%)
***Archangium* sp. WIMLSP1**	13.0	150	1	10 867	68.8	99.8 (*Archangium gephyra*)	97.2 (*A. violaceum*)
***Microvirga* sp. WIMLSP1**	3.9	40	1	3840	61.2	99.3 (*Microvirga* sp. GI_Sw3_5a_4)	84.4% (*Microvirga solisilvae*)
***Archangium* sp. WIMLSP2**	12.9	150	1	10 737	68.7	99.8 (*A. gephyra*)	97.2 (*A. violaceum*)
***Microvirga* sp. WIMLSP2**	4.3	40	1	3840	61.2	99.3 (*Microvirga* sp. GI_Sw3_5a_4)	82.0 (*Mi. solisilvae*)
***Archangium* sp. FLWO**	14.0	40	1	11 642	68.1	99.8 (*Archangium lansingense*)	96.4 (*A. lansingense*)
***Microvirga* sp. FLWO**	3.8	220	1	3658	60.9	99.1 (*Microvirga* sp. GI_Sw3_5a_4)	84.5 (*Mi. solisilvae*)
***Cystobacter* sp. DLMAZ**	12.0	84	1	10 528	68.5	99.9 (*Cystobacter fuscus*)	99.3 (*Cystobacter ferrugineus*)
***Microvirga* sp. DLMAZ**	4.1	351	1	4001	61.4	99.0 (*Microvirga* sp. MB12)	81.9 (*Mi. solisilvae*)

**Figure 2:**
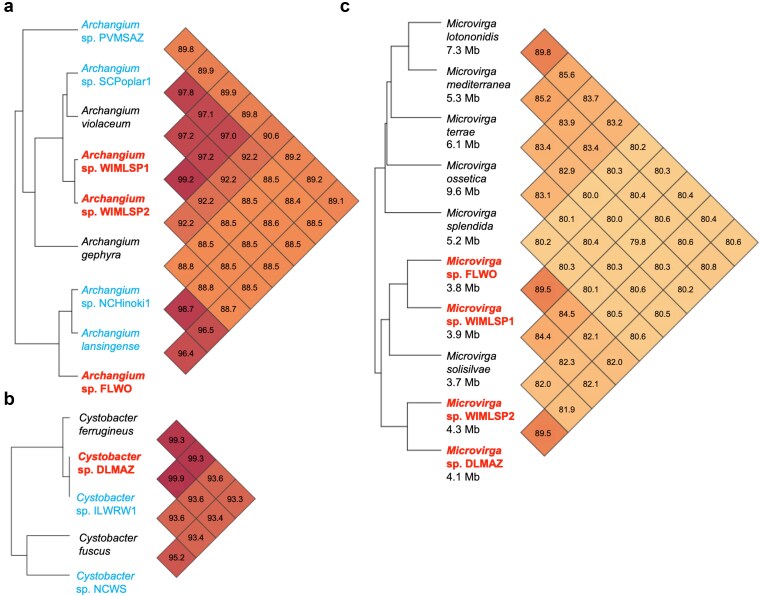
Average nucleotide identity heatmaps for *Archangium* (a), *Cystobacter* (b), and *Microvirga* (c). MAGs from swarm consortia (red) compared with type strain representatives (black) and environmental isolates (blue). Genome sizes for *Microvirga* are included to denote the differences in genome sizes between monoculture and swarm consortium–associated *Microvirga*. Heatmaps generated using OAT [46].

Utilizing ANI and dDDH values according to established methods for taxonomic placement [61, 62], we determined *Archangium* present in WIMLSP1 and WIMLSP2 share 99.2% ANI, and both are sub-species of the type strain *A. violaceum* with ~97.2% ANI. *Archangium* sp. FLWO is a sub-species of the type strain *A. lansingense* (96.4% ANI), and *Cystobacter* sp. DLMAZ shares 99.3% ANI with the sequenced strain *Cystobacter ferrugineus* and 93.4% ANI with the type strain *Cy. fuscus*. None of the myxobacterial MAGs met the established thresholds to be considered novel species (Fig. 2a and b).

All companion *Microvirga* are candidate novel species with <85% ANI shared between sequenced MAGs and the most similar type strain, *Microvirga solisilvae* [63]. These ANI values are well below the established threshold (<95% ANI) to be considered a novel species. *Microvirga* from each swarm consortium share <90% ANI and appear to be distinct species, including *Microvirga* sp. WIMLSP1 and *Microvirga* sp. WIMLSP2. From these results, we determined that WIMLSP1 and WIMLSP2 are different swarm consortia with highly related *Archangium* spp. (99.2% ANI) and much less related *Microvirga* spp. (82.3% ANI). Other than the closest relative type strain species, *Mi. solisilvae*, swarm consortium–associated *Microvirga* have much smaller genomes (~3.7–4 Mb) than sequenced, monocultured *Microvirga* (5.2–9.6 Mb) (Fig. 2c). From these observations, we hypothesize that symbiosis in swarm communities may have contributed to *Microvirga* gene loss over time resulting in genome reduction.

### Comparative genomics indicate horizontal gene transfer in swarm communities

**Figure 3:**
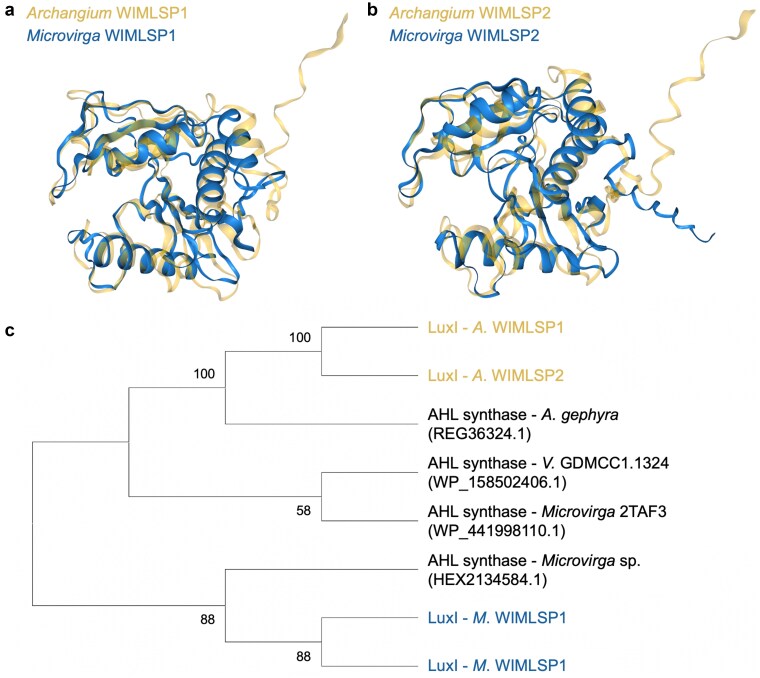
Alignment of structural models for AHL synthases present in each member of WIMLSP1 (a) and WIMLSP2 (b) swarm consortia. Evolutionary analysis of myxobacterial and microvirgal AHL synthases (c) from a bootstrap consensus tree inferred from 300 replicates. Values shown next to branches are the percentage of replicated trees with the associated taxa clustered together. Structural models of AHL synthases were generated using the AlphaFold Server and aligned with FoldMason MSA [55, 56]. Evolutionary history was inferred using the Maximum Likelihood method and JTT matrix–based model in MEGA X [54, 99, 100].

Provided MAG sequence data for each consortium strain, we sought additional attributes of swarm communities that would be indicative of symbiosis. The JGI IMG/MER database was used to analyze the phylogenetic distribution of genes from consortia metagenomes to assess potential horizontal gene transfer within swarm consortia [42, 64] (Tables S8 and S9). Using this approach, LuxI-like AHL synthases homologous to *Microvirga* were identified in *Archangium* MAGs from WIMLSP1 and WIMLSP2 (Table S9) [65, 66]. Both LuxI homologs share >70% amino acid identity with an AHL synthase from *Microvirga* sp. 2TAF3. LuxI-like AHL synthases from *A.* WIMLSP1 and *A.* WIMLSP2 were also highly homologous to the only known myxobacterial AHL synthases previously discovered from *A. gephyra* (>90% identity) and *Vitiosangium* sp. GDMCC 1.1324 (>70% identity) [67]. LuxI homologs were also present in *Microvirga* MAGs from WIMLSP1 and WIMLSP2. Foldseek analysis of cognate LuxI structures from each swarm consortium reveal structural similarity (Fig. 3a and 3b), and phylogenetic analysis corroborates a shared evolutionary history between *Archangium* and *Microvirga* AHL synthases (Fig. 3c) [55, 56]. These results suggest that horizontal transfer of an AHL synthase gene from *Microvirga* may account for the AHL synthases present in *A.* WIMLSP1, *A.* WIMLSP2, *A. gephyra*, and *V.* GDMCC 1.1324.

**Figure 4:**
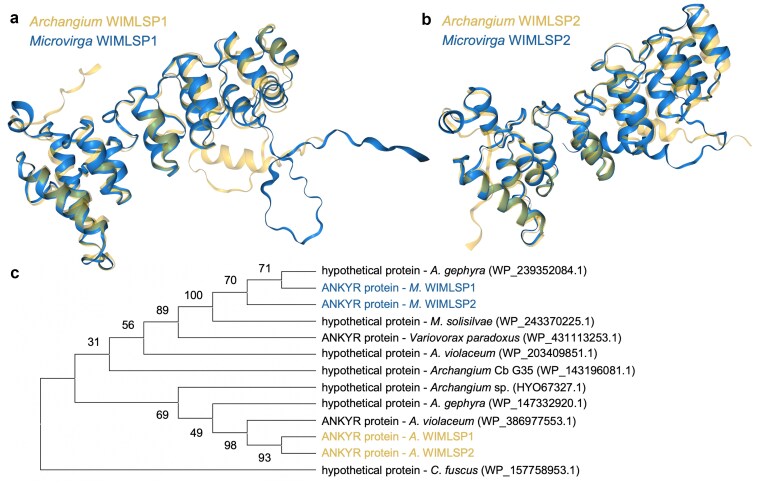
Alignment of structural models for ANKYR proteins present in each member of WIMLSP1 (a) and WIMLSP2 (b) swarm consortia. Evolutionary analysis of myxobacterial and microvirgal ANKYR proteins (c) from a bootstrap consensus tree inferred from 300 replicates. Values shown next to branches are the percentage of replicated trees with the associated taxa clustered together. Structural models of AHL synthases were generated using the AlphaFold Server and aligned with FoldMason MSA [55, 56]. Evolutionary history was inferred using the Maximum Likelihood method and Le_Gascuel_2008 model in MEGA X [54, 99, 100].

Proteins with homology to myxobacterial proteins were found in all *Microvirga* MAGs. All *Microvirga* MAGs include encoded proteins homologous with *Archangium* proteins (Table S10), including *Microvirga* sp. DLMAZ which has no gene products that share homology with sequenced *Cystobacter*. Subsequent analysis revealed homologous ankyrin repeat domain-containing (ANKYR) proteins present in all members of WIMLSP1 and WIMLSP2 swarm consortia (Table S10). A sequence similarity network (SSN) generated from the shared ANKYR proteins using the Enzyme Function Initiative Enzyme Similarity Tool (EFI-EST) identified 15 homologous entries in the UniProt database (Fig. S5) [57, 59]. Homologous ANKYR proteins in the generated SSN are exclusively present in *Archangiaceae* within the phylum *Myxococcota*. EFI-EST analysis identified ANKYR proteins variably present in eight genera from the phylum *Pseudomonadota*, and none were from the genus *Microvirga*. However, comparative genomic analysis of the 157 sequenced *Microvirga* available at the National Center for Biotechnology and Information (NCBI) genome database revealed the presence of ANKYR protein homologs in just two additional *Microvirga, Mi. solisilvae* and *Microvirga* sp. ACRRW. The spatial organization of ANKYR genes from *Archangium* and *Microvirga* spp. was analyzed using the Enzyme Function Initiative Genome Neighborhood Tool (EFI-GNT). ANKYR encoding genes from *Archangium* were all located immediately downstream of evolved VrgG genes associated with the type 6 secretion systems (T6SSs). Signal peptide sequences were subsequently detected in all ANKYR proteins from WIMLSP1 and WIMLSP2 consortia members using SignalP-6.0. Proximity to T6SSs in *Archangium* and the presence of signaling peptides indicate that ANKYR proteins could have a role in T6SS-associated toxicity or resistance mechanisms. Comparing the spatial organization of ANKYR encoding genes for *Mi. solisilvae, Mi.* sp. ACRRW, *Mi.* WIMLSP1, and *Mi.* WIMLSP2, we observed a neighboring XerC tyrosine recombinase in each genome (Fig. S6). XerC-dependent phage integration has been previously reported [68–70], and proximity to all ANKYR-encoding genes present in sequenced *Microvirga* buttresses the likelihood of horizontal acquisition. ANKYR proteins from WIMLSP1 and WIMLSP2 also share high nucleotide identities with >65% coverage values (Table S10; Fig. S7), modeled structural homology (Fig. 4a and b), and evolutionary histories supported by phylogenetic analysis (Fig. 4c). Taken together, our data suggest horizontal transfer of ANKYR proteins from *Archangium* to *Microvirga* in WIMLSP1 and WIMLSP2.

### Auxotrophies and potential metabolic exchanges in swarm consortia identified by genome-scale metabolic modeling

Metabolic exchanges in consortia that alleviate auxotrophies of members would also indicate potential for interbacterial symbiosis in swarm communities [71]. Genome-scale metabolic models (GEMs) were built for consortia MAGs and related monoculture species for comparison using the ModelSEED2 pipeline and subsequently characterized to predict auxotrophy (Tables 2 and S11) [47–49]. All swarm consortia MAGs were secondarily analyzed with GapMind to corroborate predicted amino acid auxotrophies [50]. All *Archangium* consortium members are predicted to be branched-chain amino acid (BCAA) auxotrophs as well as L-histidine, folate, and riboflavin auxotrophs (Table S11). Myxobacteria are often BCAA auxotrophs that require acquisition of BCAAs from prey lysates [72]. Comparing consortium *Archangium* with sequenced, monocultured *Archangium*, these appear to be somewhat general auxotrophies within the genus. A similar comparison of consortium and monoculture *Cystobacter* revealed no common auxotrophies within the genus, and *C.* DLMAZ was found to only share L-histidine auxotrophy with analyzed *Archangium*. Comparative analysis of consortia metagenomes using assigned KEGG orthologies confirmed the absences of dihydroxy-acid dehydratase *ilvD*, 2-isopropylmalate synthase *leuA*, and citramalate isomerase subunits *leuCD* required for BCAA biosynthesis in *Archangium* from WIMLSP1, WIMLSP2, and FLWO (Fig. S8) [51–53, 73].

**Table 2:** Auxotrophies of consortium-associated *Microvirga* (bolded) and monoculture *Microvirga*.

	L-His	L-Met	Folate	Pantothenate	Riboflavin	Spermidine
***Microvirga* sp. WIMLSP1**	P	A^a^	A	A	A	A
***Microvirga* sp. WIMLSP2**	P	A^a^	A	P	A	A
***Microvirga* sp. FLWO**	P	A^a^	A	A	A	A
***Microvirga* sp. DLMAZ**	A^b^	A^a^	A	P	A	A
*Microvirga solisilvae*	P	P	A	A	P	A
*Microvirga farnensis*	P	P	A	P	P	A
*Microvirga* sp. 17 mud 1–3	P	P	A	P	P	A
*Microvirga ossetica*	P	P	A	P	P	A
*Microvirga subterranea*	P	P	A	P	P	A
*Microvirga terrae*	P	P	A	P	P	P
*Microvirga lotononidis*	P	P	P	P	P	P

^a^L-methionine auxotrophy in *Microvirga* was predicted by metabolic models but was not observed in Gapmind analysis [50].

^b^Auxotrophies predicted by GapMind analysis but not metabolic models.

Predicted auxotrophies of consortium-associated and monoculture *Microvirga* were more varied with minimal overlap in auxotrophies between strains (Table 2). All four consortium-associated *Microvirga* were predicted to be L-methionine, folate, and riboflavin auxotrophs from GEM analysis. However, subsequent Gapmind analysis predicted all consortium *Microvirga* to be methionine prototrophs with medium confidence. None of the analyzed monoculture *Microvirga* were predicted to be L-methionine auxotrophs by either GEM or Gapmind analysis. Methionine is considered a biosynthetically costly amino acid, and methionine auxotrophy has been found to promote metabolic exchanges between symbionts [74]. Using KEGG orthologies to assess predicted L-methionine auxotrophy, we confirmed the absence of the homoserine *O*-succinyltransferase *metA* required for L-methionine biosynthesis via L-cystathionine in all four consortium-associated *Microvirga* [75]. Alternatively, all four *Microvirga* were found to have a complete sulfhydrylation pathway that utilizes hydrogen sulfide for L-methionine production [76]. Discrepancies between GEM analysis and Gapmind analysis are likely due to the intrinsic homology between homoserine *O-*succinyltransferase *metA* from the L-cystathionine-dependent methionine biosynthetic pathway and *metX* from the sulfhydration pathway. Intrigued by this observation, we verified the absence of an assimilatory sulfate reduction pathway in all four *Microvirga* to exclude potential L-methionine prototrophy via direct sulfhydrylation of *O*-acetyl-L-homoserine to L-homocysteine [77]. These observations were mirrored in consortium-associated *Archangium* with no observed *metA* orthologs. However, complete direct sulfhydrylation L-methionine biosynthetic pathways are present in all four *Archangium*.

**Figure 5:**
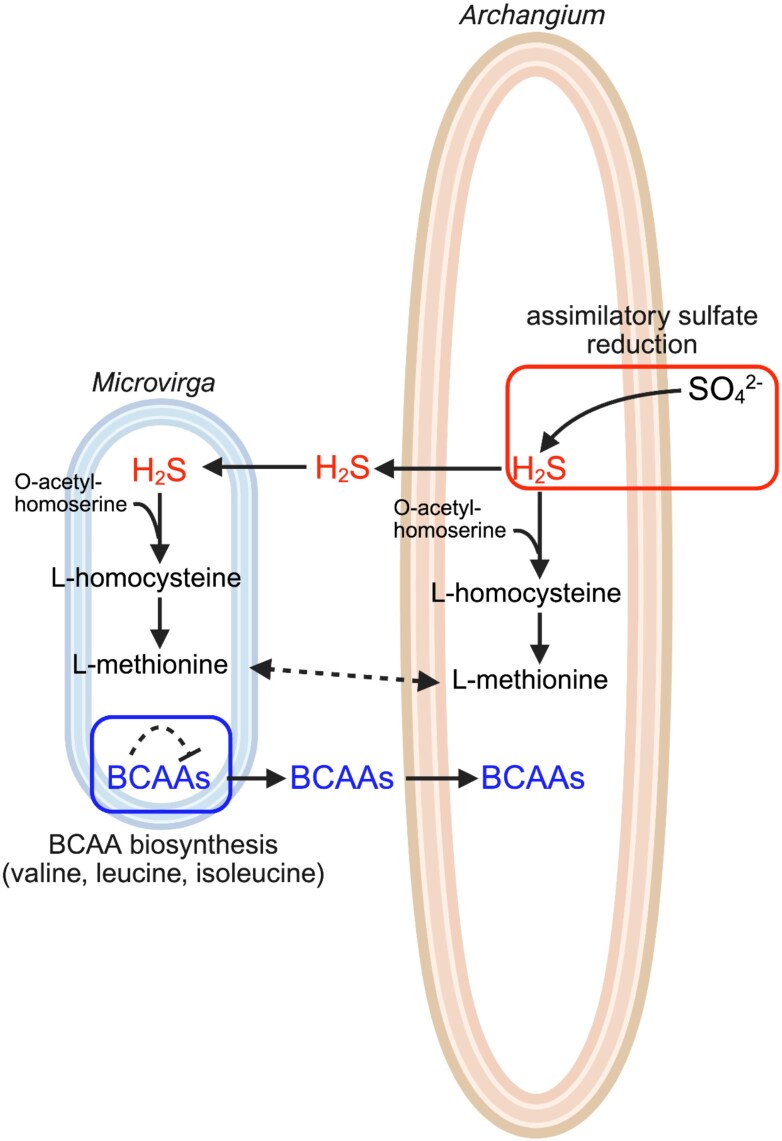
Proposed metabolic exchanges in WIMLSP1 and WIMLSP2 to alleviate L-methionine auxotrophy in *Microvirga* and BCAA auxotrophy in *Archangium*. Image generated in BioRender.

Identification of potential nutrient exchanges in swarm consortia suggests consortia stability and support interbacterial symbiosis. Exchange of BCAAs from *Microvirga* to *Archangium* provides a straightforward opportunity for cross-feeding in swarm consortia (Fig. 5). Accumulation of BCAAs and various intermediate metabolites has been shown to inhibit BCAA biosynthesis [78], and BCAA consumption by *Archangium* would promote BCAA biosynthesis in *Microvirga*. The overlap in direct sulfhydrylation L-methionine biosynthetic pathways between members provided an additional potential nutrient exchange in each consortium. Hydrogen sulfide exchange from producing *Archangium*, via intact assimilatory sulfate reduction pathways, would enable L-methionine biosynthesis from *Microvirga* in swarm (Fig. 5). Biosynthesis of L-methionine by consortia *Archangium* may also provide L-methionine directly to consortia *Microvirga* independent of hydrogen sulfide exchange. These results provide potential nutrient exchanges between members of swarm consortia that would alleviate essential amino acid auxotrophies.

### Intraconsortium predation of *Microvirga* companions is a rare occurrence

**Figure 6:**
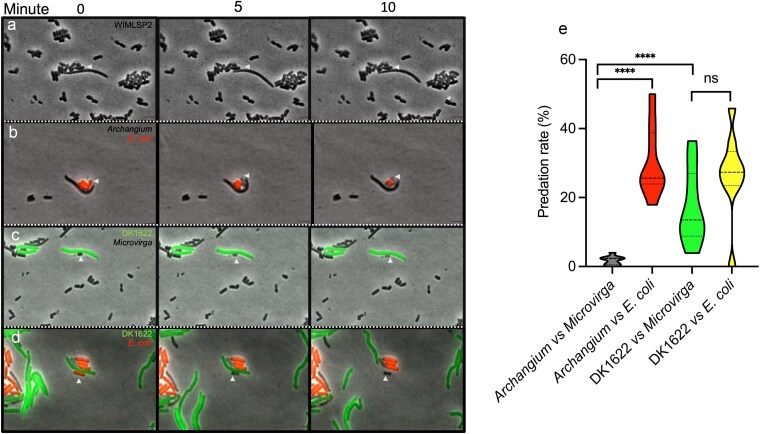
Contact-dependent predation of bacterial prey by myxobacteria. Time-lapse microscopy stills demonstrate predator–prey interactions at 0, 5, and 10 min following initial contact (white arrowheads indicate prey cells). (a) *Archangium* WIMLSP2 interacting with *Microvirga* WIMLSP2 cells. (b) *Archangium* WIMLSP2 interacting and killing *E. coli* cells. (c) *My. xanthus* DK1622 interacting with and killing *Microvirga* WIMLSP2. (d) *My. xanthus* DK1622 interacting with and killing *E. coli*. (e) Violin plots illustrating the distributions of predation rates (%) calculated from time-lapse movies for each predator–prey pairing. Each violin represents the full distribution of predation rates, with the dashed lines indicating the median and dotted lines indicating the interquartile range. Statistical comparisons were performed using a two-tailed Mann–Whitney *U* test. **** indicated *P* < .0001; ns, not significant. See corresponding Movies S1–S4 for additional details.

Predatory lifestyles of myxobacteria in the class *Myxococcia*—including *Archangium—*complicate the possibility of stable symbiosis within swarm consortia, as myxobacteria are known to exhibit exceptional predatory capacities. Predation of *Alphaproteobacteria* such as *Sinorhizobium meliloti* and *Agrobacterium tumefaciens* has been documented [79, 80]. To assess if predation occurs within a naturally occurring consortia, we leveraged distinct cell-size differences between members and selected the WIMLSP2 consortium as the best model consortium due to the favorable ratio of members observed previously by SEM. Time-lapse movies of WIMLSP2 were recorded, and cell–cell interactions between consortium members from WIMLSP2 were enumerated to record the number of times that *Mi.* WIMLSP2 cells lysed following contact with *A.* WIMLSP2. Of the 1299 observed cell–cell interactions, only 23 resulted in *Microvirga* lysis, yielding an intraconsortium predation rate of just 2.5% (Fig. 6a). These results indicate that predation of *Microvirga* companions by *Archangium* was rare within the swarm consortium WIMLSP2, supporting the interpretation that these species coexist stably rather than primarily engaging in predator–prey interaction.

### Confirmed predation of *E. coli* by swarm consortium

We revisited the predatory capacity of WIMLSP2 using *E. coli* as prey to test whether the low intraconsortium predation rate reflected *bona fide* resistance by *Microvirga* rather than a defect in *Archangium* predation. Although all swarm consortia were originally isolated using prey baiting with *E. coli*, we sought to enumerate consortium predation with a mCherry-labeled *E. coli* prey. Fluorescent *E. coli* cells enabled us to similarly track *A.* WIMLSP2 contact with *E. coli* and document subsequent lysis events. A total of 139 lysis events were observed after monitoring 502 cell–cell interactions between *A.* WIMLSP2 and introduced *E. coli* prey. The calculated 28% predation rate from these results demonstrate that WIMLSP2 capably predates *E. coli* that is significantly different than the *Microvirga* lysis rate (Fig. 6b). For comparison, we performed the same assay using *My. xanthus* DK1622-GFP and *E. coli*–mCherry and observed 278 lysis events out of 930 total cell–cell interactions, confirming the expected predatory efficiency of the model myxobacterium *My. xanthus* DK1622 [81]. These results show *A.* WIMLSP2 and *My. xanthus* have comparable *E. coli* predation rates (28% and 30%) and suggest that *Microvirga* swarm companions may be resistant to myxobacteria predation.

### *My. xanthus* predates *Microvirga* companions

Using the same approach to determine the predation rate of *Microvirga* WIMLSP2 by *My. xanthus* DK1622-GFP, we monitored 1339 total predator–prey interactions and recorded 199 lysis events. From these results, a predation rate of 15% was calculated (Fig. 6c). This rate is higher than that observed for predation by *A.* WIMLSP2, indicating that *Mi.* WIMLSP2 is more susceptible to DK1622-GFP than to its native myxobacterial partner.

## Discussion

Our discovery of myxobacterial swarm consortia corroborates the myxobacterial companionships reported nearly three decades ago [36, 37]. Phylogenetic differences between myxobacteria present in each reported swarm consortium, with *Chondromyces* from the subclass *Polyangiaa* and *Archangium* from the subclass *Myxococcia*, suggest interbacterial symbiosis with myxobacteria may be prevalent in polymicrobial communities. Supporting the potential prevalence of swarm consortia, a recent investigation of biotic interactions in the alpine soil microbiome during seasonal shifts observed positive associations between *Alphaproteobacteria* and *Myxococcota* [82]. Alphaproteobacterial genomes have extraordinary plasticity associated with facultative, intracellular, and free-living lifestyles reported from the class, and horizontal gene transfer occurs commonly in *Alphaproteobacteria* [83]. Genetic adaptability is reflected in the substantial differences in alphaproteobacterial genome sizes, ranging from 1 to 9 Mb. The lifestyle differences of *Microvirga* exemplify the genetic variability of *Alphaproteobacteria* [84]. The genus currently has 27 species with valid descriptions that have been isolated from a range of environments other than soil including the atmosphere, hot springs, deep-sea thermal aquifers, plant root nodules, metal industry waste, and human stool. Described *Microvirga* strains also include plant and human symbiotes demonstrating precedence for symbiosis in the genus [84]. *Microvirga* from swarm consortia appear to be novel species. Consortium-associated *Microvirga* are the first reported *Microvirga* found to be interbacterial symbionts and the second reported “companions” of myxobacteria. Noted metabolic exchanges and horizontally transferred AHL synthases and ANKYR gene products are indications of symbiosis.

Although exogenous AHLs have been shown to elicit responses from myxobacteria, the underlying mechanisms remain unknown, and myxobacteria are not known to participate in AHL-mediated quorum sensing [85, 86]. There are no documented instances of a complete LuxR/LuxI quorum sensing system in a myxobacterium. The previously mentioned functional AHL synthases from *A. gephyra* and *V.* GDMCC 1.1324 are orphaned without a paired LuxR receptor, and AHL production has not been directly observed from either myxobacterium [67]. Each myxobacterial LuxI-homolog required heterologous expression in an *E. coli* host to confirm functionality and biosynthesis of AHLs. Our data suggest that AHL synthases from *A.* WIMLSP1 and *A.* WIMLSP2 are highly similar to the *A. gephyra* and *V.* GDMCC 1.1324 AHL synthases. Horizontal acquisition of biosynthetic genes has been well documented in myxobacteria [20, 87], and phylogenetic analysis supports a potential shared evolutionary history between AHL synthases from myxobacteria and *Microvirga*. Production of AHLs by myxobacteria was previously speculated to benefit predation by influencing prey AHL quorum sensing and undermining prey coordination [67]. Reduced predation of companion *Microvirga* in WIMLSP2 implies a potential alternative utility for myxobacterial production of AHLs in consortia. Production of AHLs by *Archangium* in consortia may influence numerous microvirgal characteristics including growth, and we are intrigued to further explore quorum sensing in swarm consortia.

ANKYR proteins have been implicated in host immune avoidance in a variety of symbiotic relationships. As examples, ANKYR proteins VAPYRIN and IGN1 are required for host accommodation in fungal– and bacterial–plant symbioses [88, 89], and ANKYR genes of the intracellular *Wolbachia* symbionts rapidly evolve and have been suggested to contribute to *Wolbachia*–*Drosophila* symbiosis [90–92]. Proximity to T6SSs in *Archangium* along with all of the mentioned ANKYR proteins containing signal sequences, suggests a secretory role for them. Proteins with ankyrin repeat domains have been associated with secreted effectors in type 4 section systems and engineered to function as antitoxins against *Clostridium difficile* toxin B [93, 94]. Potential functionality as anti-toxins associated with *Archangium* T6SSs would align with horizontal acquisition of ANKYR genes benefiting *Microvirga* companions and may account for reduced predation observed in WIMLSP2. In future work, we seek to develop genetic approaches for swarm consortium engineering, which will enable deeper investigations of ANKYR proteins shared by members of WIMLSP1 and WIMLSP2 to explore potential contributions to myxobacterial symbiosis.

The presence of distinguishable microvirgal symbionts, *Mi.* WIMLSP1 and *Mi.* WIMLSP2, associated with practically the same strain of *Archangium* from the same soil sample, suggests that symbiosis in swarm consortia is not exclusive and likely facultative. We previously isolated monocultures of *Archangium* sp. SCPoplar1, *A. lansingense*, and *Cystobacter* sp. ILWRW that are nearly identical (97.0%, 96.4%, and 99.9% ANI, respectively) to myxobacteria from swarm consortia [20, 87]. We hypothesize *Microvirga* that have adapted to avoid predation to certain myxobacterial, establish transient symbiotic relationships with them in polymicrobial communities and benefit from nutrients released by myxobacterial lysis of prey. *Microvirga* have been noted to be common “contaminates” that complicate or prevent successful isolation of axenic myxobacteria from soil [95]. Our observations that support this hypothesis include genes phylogenetically associated with a variety of myxobacteria present in genomes of consortium-associated *Microvirga* (Table S8) and the genetic variability of consortium-associated *Microvirga*. For example, the BNR-like beta propeller repeat proteins from *Mi.* WIMLSP1 and *Mi.* DLMAZ with homologs in *Archangium* (Table S10) only share 55% amino acid identity and may have been acquired from previous myxobacterial symbionts. The DLMAZ swarm consortium potentially reflects the proposed transient existence of a swarm consortium in nature. The only swarm consortium found to not include an *Archangium*, DLMAZ has no shared genetic features that may have passed horizontally between *Cystobacter* and *Microvirga*, and *C.* DLMAZ is not predicted to be a BCAA auxotroph. Adaptation of *Mi.* DLMAZ in a previous swarm consortium and loss of myxobacterial companion provides a potential explanation for discrepancies between it and other consortia. However, without further knowledge of the features involved, myxobacterial conditioning of symbionts and their movement between myxobacteria symbionts remain hypothetical.

Our findings demonstrate that *A*. WIMLSP2 exhibits reduced predation toward its companion *Mi*. WIMLSP2 while retaining robust predatory capacity against nonsymbiotic prey. This attenuation is not attributable to a generalized defect in killing, as both *A*. WIMLSP2 and the model myxobacterium *My. xanthus* DK1622 displayed comparable predation efficiencies against *E. coli* (28% and 30%, respectively). Instead, the reduced susceptibility of *Microvirga* appears specific to its interaction with *Archangium*, consistent with an active, partner-specific modulation of predatory behavior. Although the molecular basis of this protection remains unresolved, myxobacteria are known to rely on cell-surface recognition systems to regulate social interactions, including kin discrimination and cooperative behaviors [3, 5]. One possibility is that *Microvirga* presents surface features that are recognized by *Archangium*, thereby dampening the initiation or execution of predation. Alternatively, *Microvirga* may produce inhibitory factors, such as antitoxins or signaling molecules, which interfere with downstream killing mechanisms following contact. Distinguishing between recognition-based avoidance and biochemical inhibition will be essential for defining how this interaction is regulated. More broadly, these findings suggest that myxobacterial predation can be selectively tuned toward particular neighbors, providing a potential mechanism by which stable associations and cooperative behaviors emerge within otherwise antagonistic microbial communities.

Previously described proto-farming of bacterial symbionts by the predatory, social amoeba *Dictystelium discoideum* serves as an established example of farming symbiosis between microbial predators and prey [96, 97]. A farming phenotype present in natural isolates of *D. discoideum* that demonstrates bacterial husbandry has been reported [96]. Farming *D. discoideum* clones curtail predation of bacteria and utilize fruiting body formation and subsequent sporulation to carry and seed bacterial crops during spore dispersal, and access to the dispersed food source in the absence of bacterial prey benefits farmers [96–98]. Access to microvirgal cells for nutrition in the absence of suitable prey would similarly benefit myxobacteria in swarm communities. Other parallels between farming symbiosis in *D. discoideum* and myxobacterial swarm consortia include the participation of social, cooperative predators with reduced predation of symbionts. Access to cultivable swarm consortia provides the opportunity to further investigate potential symbiotic farming by myxobacteria.

## Conclusion

*Myxococcota* are a ubiquitous, keystone taxon in soil community structure with an outsized impact on nutrient cycling in the soil food web. Our results afford foundational insight into interbacterial symbiosis attributable to phenotypic selection of predation-resistant populations in polymicrobial communities. Discovery of a single strain of *Archangium* with two different microvirgal companions from the same soil sample suggests swarm consortia members are facultative symbionts. Although we have not determined if any swarm consortium members are facultative or obligate symbionts, we suspect obligate symbiosis in swarm consortia to be a potential outcome in nature that may contribute to discrepancies between monocultured myxobacteria and myxobacteria present in metagenomic analysis of soil. Continued investigation of swarm consortia and comparison of xenic and axenic myxobacterial cultures will expand the current understanding of myxobacterial symbiosis, development, metabolism, and predation in polymicrobial communities.

## Supplementary Material

Supplementary_material_wrag140

## Data Availability

The data sets presented in this study can be found in online repositories. JGI Genomes Online Database (GOLD) Analysis Project IDs are as follows: WIMLSP1 (Ga0669685), WIMLSP2 (Ga0646599), FLWO (Ga0654980), and DLMAZ (Ga0646598).

## References

[1] Munoz-Dorado J, Marcos-Torres FJ, Garcia-Bravo E et al. Myxobacteria: moving, killing, feeding, and surviving together. *Front Microbiol* 2016;7:781. 27303375 10.3389/fmicb.2016.00781 PMC4880591

[2] Perez J, Moraleda-Munoz A, Marcos-Torres FJ et al. Bacterial predation: 75 years and counting! *Environ Microbiol* 2016;18:766–79. 10.1111/1462-2920.13171 26663201

[3] Cao P, Wall D. Direct visualization of a molecular handshake that governs kin recognition and tissue formation in myxobacteria. *Nat Commun* 2019;10:3073. 10.1038/s41467-019-11108-w 31300643 PMC6626042

[4] Cossey SM, Yu YN, Cossu L et al. Kin discrimination and outer membrane exchange in *myxococcus xanthus*: experimental analysis of a natural population. *PLoS One* 2019;14:e0224817. 10.1371/journal.pone.0224817 31774841 PMC6880969

[5] Sah GP, Wall D. Kin recognition and outer membrane exchange (OME) in myxobacteria. *Curr Opin Microbiol* 2020;56:81–8. 10.1016/j.mib.2020.07.003 32828979 PMC7744404

[6] Dai W, Wang N, Wang W et al. Community profile and drivers of predatory myxobacteria under different compost manures. *Microorganisms* 2021;9:2193. 10.3390/microorganisms9112193 PMC8622275 34835319

[7] Findlay BL . The chemical ecology of predatory soil bacteria. *ACS Chem Biol* 2016;11:1502–10. 10.1021/acschembio.6b00176 27035738

[8] Livingstone PG, Morphew RM, Whitworth DE. Myxobacteria are able to prey broadly upon clinically-relevant pathogens, exhibiting a prey range which cannot Be explained by phylogeny. *Front Microbiol* 2017;8:1593. 10.3389/fmicb.2017.01593 28878752 PMC5572228

[9] Mohr KI . Diversity of myxobacteria-we only see the tip of the iceberg. *Microorganisms* 2018;6:84. 10.3390/microorganisms6030084 PMC6164225 30103481

[10] Wang C, Lv Y, Li A et al. Culture-dependent and -independent methods revealed an abundant myxobacterial community shaped by other bacteria and pH in Dinghushan acidic soils. *PLoS One* 2020;15:e0238769. 10.1371/journal.pone.0238769 32925929 PMC7489521

[11] Zhang L, Dong C, Wang J et al. Predation of oomycetes by myxobacteria via a specialized CAZyme system arising from adaptive evolution. *ISME J* 2023;17:1089–103. 10.1038/s41396-023-01423-y 37156836 PMC10284895

[12] Zhang L, Lueders T. Micropredator niche differentiation between bulk soil and rhizosphere of an agricultural soil depends on bacterial prey. *FEMS Microbiol Ecol* 2017;93:fix103. 10.1093/femsec/fix103 28922803

[13] Zhou XW, Li SG, Li W et al. Myxobacterial community is a predominant and highly diverse bacterial group in soil niches. *Environ Microbiol Rep* 2014;6:45–56. 10.1111/1758-2229.12107 24596262

[14] Zhou Y, Zhang X, Yao Q et al. Both soil bacteria and soil chemical property affected the micropredator Myxobacterial community: evidence from natural Forest soil and greenhouse rhizosphere soil. *Microorganisms* 2020;8:8. 10.3390/microorganisms8091387 PMC7563646 32927762

[15] Petters S, Gross V, Sollinger A et al. The soil microbial food web revisited: predatory myxobacteria as keystone taxa? *ISME J* 2021;15:2665–75. 10.1038/s41396-021-00958-2 33746204 PMC8397742

[16] Bader CD, Panter F, Muller R. In depth natural product discovery - Myxobacterial strains that provided multiple secondary metabolites. *Biotechnol Adv* 2020;39:107480. 10.1016/j.biotechadv.2019.107480 31707075

[17] Gregory K, Salvador LA, Akbar S et al. Survey of biosynthetic gene clusters from sequenced myxobacteria reveals unexplored biosynthetic potential. *Microorganisms* 2019;7:181. 10.3390/microorganisms7060181 PMC6616573 31238501

[18] Herrmann J, Fayad AA, Muller R. Natural products from myxobacteria: novel metabolites and bioactivities. *Nat Prod Rep* 2017;34:135–60. 10.1039/C6NP00106H 27907217

[19] Landwehr W, Wolf C, Wink J. Actinobacteria and myxobacteria-two of the most important bacterial resources for novel antibiotics. *Curr Top Microbiol Immunol* 2016;398:273–302. 10.1007/82_2016_503 27704272

[20] Ahearne A, Phillips K, Knehans T et al. Chromosomal organization of biosynthetic gene clusters, including those of nine novel species, suggests plasticity of myxobacterial specialized metabolism. *Front Microbiol* 2023;14:1227206. 10.3389/fmicb.2023.1227206 37601375 PMC10435759

[21] Baltz RH . Gifted microbes for genome mining and natural product discovery. *J Ind Microbiol Biotechnol* 2017;44:573–88. 10.1007/s10295-016-1815-x 27520548

[22] Baltz RH . Genome mining for drug discovery: progress at the front end. *J Ind Microbiol Biotechnol* 2021;48:kuab044. 10.1093/jimb/kuab044 PMC8788784 34279640

[23] Mohr KI, Stechling M, Wink J et al. Comparison of myxobacterial diversity and evaluation of isolation success in two niches: Kiritimati Island and German compost. *Microbiologyopen* 2016;5:268–78. 10.1002/mbo3.325 26669488 PMC4831471

[24] Phillips KE, Akbar S, Stevens DC. Concepts and conjectures concerning predatory performance of myxobacteria. *Front Microbiol* 2022;13:1031346. 10.3389/fmicb.2022.1031346 36246230 PMC9556981

[25] Wang J, Wang J, Wu S et al. Global geographic diversity and distribution of the myxobacteria. *Microbiol Spectrum* 2021;9:e0001221. 10.1128/Spectrum.00012-21 PMC8552515 34259548

[26] Nair RR, Velicer GJ. Predatory bacteria select for sustained prey diversity. *Microorganisms* 2021;9:2079. 10.3390/microorganisms9102079 PMC8540638 34683400

[27] Nair RR, Vasse M, Wielgoss S et al. Bacterial predator-prey coevolution accelerates genome evolution and selects on virulence-associated prey defences. *Nat Commun* 2019;10:4301. 10.1038/s41467-019-12140-6 31541093 PMC6754418

[28] Akbar S, Stevens DC. Functional genomics study of *pseudomonas putida* to determine traits associated with avoidance of a myxobacterial predator. *Sci Rep* 2021;11:16445. 10.1038/s41598-021-96046-8 34385565 PMC8360965

[29] Contreras-Moreno FJ, Moraleda-Munoz A, Marcos-Torres FJ et al. Siderophores and competition for iron govern myxobacterial predation dynamics. *ISME J* 2024;18. 10.1093/ismejo/wrae077 PMC11388931 38696719

[30] Lee N, Kim W, Chung J et al. Iron competition triggers antibiotic biosynthesis in *Streptomyces coelicolor* during coculture with *myxococcus xanthus*. *ISME J* 2020;14:1111–24. 10.1038/s41396-020-0594-6 31992858 PMC7174319

[31] Perez J, Munoz-Dorado J, Brana AF et al. *Myxococcus xanthus* induces actinorhodin overproduction and aerial mycelium formation by *Streptomyces coelicolor*. *Microb Biotechnol* 2011;4:175–83. 10.1111/j.1751-7915.2010.00208.x 21342463 PMC3818858

[32] Muller S, Strack SN, Hoefler BC et al. Bacillaene and sporulation protect *Bacillus subtilis* from predation by *myxococcus xanthus*. *Appl Environ Microbiol* 2014;80:5603–10. 10.1128/AEM.01621-14 25002419 PMC4178607

[33] Muller S, Strack SN, Ryan SE et al. Identification of functions affecting predator-prey interactions between *myxococcus xanthus* and *Bacillus subtilis*. *J Bacteriol* 2016;198:3335–44. 10.1128/JB.00575-16 27698086 PMC5116937

[34] Sydney N, Swain MT, So JMT et al. The genetics of prey susceptibility to Myxobacterial predation: a review, including an investigation into *Pseudomonas aeruginosa* mutations affecting predation by *myxococcus xanthus*. *Microb Physiol* 2021;31:57–66. 10.1159/000515546 1-10. 33794538

[35] Wang C, Liu X, Zhang P et al. *Bacillus licheniformis* escapes from *myxococcus xanthus* predation by deactivating myxovirescin a through enzymatic glucosylation. *Environ Microbiol* 2019;21:4755–72. 10.1111/1462-2920.14817 31600864

[36] Jacobi CA, Assmus B, Reichenbach H et al. Molecular evidence for association between the sphingobacterium-like organism "Candidatus comitans" and the myxobacterium *Chondromyces crocatus*. *Appl Environ Microbiol* 1997;63:719–23. 10.1128/aem.63.2.719-723.1997 9023949 PMC168361

[37] Jacobi CA, Reichenbach H, Tindall BJ et al. "Candidatus comitans," a bacterium living in coculture with *Chondromyces crocatus* (myxobacteria). *Int J Syst Bacteriol* 1996;46:119–22. 10.1099/00207713-46-1-119 8573486

[38] Adaikpoh BI, Dowd SE, Stevens DC. Draft genome sequence of *Aneurinibacillus* sp. strain BA2021, isolated as a contaminant of a laboratory-cultivated predatory myxobacterium. *Microbiol Resour Announc* 2021;10:mra.00243-21. 10.1128/MRA.00243-21 PMC8086214 33927039

[39] Golding CG, Lamboo LL, Beniac DR et al. The scanning electron microscope in microbiology and diagnosis of infectious disease. *Sci Rep* 2016;6:26516. 10.1038/srep26516 27212232 PMC4876401

[40] Kolmogorov M, Yuan J, Lin Y et al. Assembly of long, error-prone reads using repeat graphs. *Nat Biotechnol* 2019;37:540–6. 10.1038/s41587-019-0072-8 30936562

[41] Nicholls SM, Quick JC, Tang S et al. Ultra-deep, long-read nanopore sequencing of mock microbial community standards. *Gigascience* 2019;8:giz043. 10.1093/gigascience/giz043 PMC6520541 31089679

[42] Chen IA, Chu K, Palaniappan K et al. The IMG/M data management and analysis system v.7: content updates and new features. *Nucleic Acids Res* 2023;51:D723–32. 10.1093/nar/gkac976 36382399 PMC9825475

[43] Freese HM, Meier-Kolthoff JP, Sarda CJ et al. TYGS and LPSN in 2025: a global Core biodata resource for genome-based classification and nomenclature of prokaryotes within DSMZ digital diversity. *Nucleic Acids Res* 2025;54:D884–91. 10.1093/nar/gkaf1110 PMC12807606 41160892

[44] Meier-Kolthoff JP, Carbasse JS, Peinado-Olarte RL et al. TYGS and LPSN: a database tandem for fast and reliable genome-based classification and nomenclature of prokaryotes. *Nucleic Acids Res* 2022;50:D801–7. 10.1093/nar/gkab902 34634793 PMC8728197

[45] Meier-Kolthoff JP, Goker M. TYGS is an automated high-throughput platform for state-of-the-art genome-based taxonomy. *Nat Commun* 2019;10:2182. 10.1038/s41467-019-10210-3 31097708 PMC6522516

[46] Lee I, Ouk Kim Y, Park SC et al. OrthoANI: an improved algorithm and software for calculating average nucleotide identity. *Int J Syst Evol Microbiol* 2016;66:1100–3. 10.1099/ijsem.0.000760 26585518

[47] Henry CS, DeJongh M, Best AA et al. High-throughput generation, optimization and analysis of genome-scale metabolic models. *Nat Biotechnol* 2010;28:977–82. 10.1038/nbt.1672 20802497

[48] Overbeek R, Olson R, Pusch GD et al. The SEED and the rapid annotation of microbial genomes using subsystems technology (RAST). *Nucleic Acids Res* 2014;42:D206–14. 10.1093/nar/gkt1226 24293654 PMC3965101

[49] Latendresse M . Efficiently gap-filling reaction networks. *BMC Bioinformatics* 2014;15:225. 10.1186/1471-2105-15-225 24972703 PMC4094995

[50] Price MN, Deutschbauer AM, Arkin AP. GapMind: automated annotation of amino acid biosynthesis. *mSystems* 2020;5:5. 10.1128/msystems.00291-20 PMC7311316 32576650

[51] Kanehisa M, Furumichi M, Sato Y et al. KEGG for taxonomy-based analysis of pathways and genomes. *Nucleic Acids Res* 2023;51:D587–92. 10.1093/nar/gkac963 36300620 PMC9825424

[52] Kanehisa M, Sato Y, Morishima K. BlastKOALA and GhostKOALA: KEGG tools for functional characterization of genome and metagenome sequences. *J Mol Biol* 2016;428:726–31. 10.1016/j.jmb.2015.11.006 26585406

[53] Ogata H, Goto S, Fujibuchi W et al. Computation with the KEGG pathway database. *Biosystems* 1998;47:119–28. 10.1016/S0303-2647(98)00017-3 9715755

[54] Kumar S, Stecher G, Li M et al. MEGA X: molecular evolutionary genetics analysis across computing platforms. *Mol Biol Evol* 2018;35:1547–9. 10.1093/molbev/msy096 29722887 PMC5967553

[55] Cameron LM, Gilchrist MM, Steinegger M. Multiple protein structure alignment at scale with FoldMason. *Science* 2026;**391**:485–488. 10.1126/science.ads6733 41610233

[56] van Kempen M, Kim SS, Tumescheit C et al. Fast and accurate protein structure search with Foldseek. *Nat Biotechnol* 2024;**42**:243–246. 10.1038/s41587-023-01773-0 PMC10869269 37156916

[57] Oberg N, Zallot R, Gerlt JA. EFI-EST, EFI-GNT, and EFI-CGFP: enzyme function initiative (EFI) web resource for genomic enzymology tools. *J Mol Biol* 2023;435:168018. 10.1016/j.jmb.2023.168018 37356897 PMC10291204

[58] Zallot R, Oberg N, Gerlt JA. The EFI web resource for genomic enzymology tools: leveraging protein, genome, and metagenome databases to discover novel enzymes and metabolic pathways. *Biochemistry* 2019;58:4169–82. 10.1021/acs.biochem.9b00735 31553576 PMC7057060

[59] Gerlt JA, Bouvier JT, Davidson DB et al. Enzyme function initiative-enzyme similarity tool (EFI-EST): a web tool for generating protein sequence similarity networks. *Biochim Biophys Acta* 2015;1854:1019–37. 10.1016/j.bbapap.2015.04.015 25900361 PMC4457552

[60] Ahearne A, Albataineh H, Dowd SE et al. Assessment of evolutionary relationships for prioritization of myxobacteria for natural product discovery. *Microorganisms* 2021;9:1376. 10.3390/microorganisms9071376 PMC8307915 34202719

[61] Chambers J, Sparks N, Sydney N et al. Comparative genomics and Pan-genomics of the Myxococcaceae, including a description of five novel species: *myxococcus eversor* sp. nov., *myxococcus llanfairpwllgwyngyllgogerychwyrndrobwllllantysiliogogogochensis* sp. nov., *myxococcus vasta*tor sp. nov., *Pyxidicoccus caerfyrddinensis* sp. nov., and *Pyxidicoccus trucidator* sp. nov. *Genome Biol Evol* 2020;12:2289–302. 10.1093/gbe/evaa212 33022031 PMC7846144

[62] Livingstone PG, Ingleby O, Girdwood S et al. Predatory organisms with untapped biosynthetic potential: descriptions of novel *Corallococcus* species *C. Aberystwythensis* sp. nov., *C. Carmarthensis* sp. nov., *C. Exercitus* sp. nov., *C. Interemptor* sp. nov., *C. Llansteffanensis* sp. nov., *C. Praedator* sp. nov., *C. Sicarius* sp. nov., and *C. Terminator* sp. nov. *Appl Environ Microbiol* 2020;86:e01931-19. 10.1128/AEM.01931-19 PMC6952226 31676482

[63] Zhang X, Feng GD, Zhen X et al. *Microvirga terricola* sp. nov. and *Microvirga solisilvae* sp. nov, isolated from forest soil. *Arch Microbiol* 2022;204:423. 10.1007/s00203-022-02963-1 35750895

[64] Mukherjee S, Stamatis D, Li CT et al. Genomes OnLine Database (GOLD) v.10: new features and updates. *Nucleic Acids Res* 2025;53:D989–97. 10.1093/nar/gkae1000 39498478 PMC11701667

[65] Fuqua C, Winans SC, Greenberg EP. Census and consensus in bacterial ecosystems: the LuxR-LuxI family of quorum-sensing transcriptional regulators. *Ann Rev Microbiol* 1996;50:727–51. 10.1146/annurev.micro.50.1.727 8905097

[66] Fuqua WC, Winans SC, Greenberg EP. Quorum sensing in bacteria: the LuxR-LuxI family of cell density-responsive transcriptional regulators. *J Bacteriol* 1994;176:269–75. 10.1128/jb.176.2.269-275.1994 8288518 PMC205046

[67] Albataineh H, Duke M, Misra SK et al. Identification of a solo acylhomoserine lactone synthase from the myxobacterium *Archangium gephyra*. *Sci Rep* 2021;11:3018. 10.1038/s41598-021-82480-1 33542315 PMC7862692

[68] Huber KE, Waldor MK. Filamentous phage integration requires the host recombinases XerC and XerD. *Nature* 2002;417:656–9. 10.1038/nature00782 12050668

[69] McLeod SM, Waldor MK. Characterization of XerC- and XerD-dependent CTX phage integration in *vibrio cholerae*. *Mol Microbiol* 2004;54:935–47. 10.1111/j.1365-2958.2004.04309.x 15522078

[70] Merino M, Acosta J, Poza M et al. OXA-24 carbapenemase gene flanked by XerC/XerD-like recombination sites in different plasmids from different *Acinetobacte*r species isolated during a nosocomial outbreak. *Antimicrob Agents Chemother* 2010;54:2724–7. 10.1128/AAC.01674-09 20385865 PMC2876395

[71] Zengler K, Zaramela LS. The social network of microorganisms - how auxotrophies shape complex communities. *Nat Rev Microbiol* 2018;16:383–90. 10.1038/s41579-018-0004-5 29599459 PMC6059367

[72] LJD Shimkets, M Dworkin, H Reichenbach. 2006. The myxobacteria. In MF Dworkin, S.; E Rosenberg; K. H Schleifer. (ed), The Prokaryotes 10.1007/0-387-30747-8_3. Springer, New York, NY.

[73] Amorim Franco TM, Blanchard JS. Bacterial branched-chain amino acid biosynthesis: structures, mechanisms, and drugability. *Biochemistry* 2017;56:5849–65. 10.1021/acs.biochem.7b00849 28977745 PMC5839172

[74] Mee MT, Collins JJ, Church GM et al. Syntrophic exchange in synthetic microbial communities. *Proc Natl Acad Sci USA* 2014;111:E2149–56. 10.1073/pnas.1405641111 24778240 PMC4034247

[75] Ferla MP, Patrick WM. Bacterial methionine biosynthesis. *Microbiology (Reading)* 2014;160:1571–84. 10.1099/mic.0.077826-0 24939187

[76] Alaminos M, Ramos JL. The methionine biosynthetic pathway from homoserine in *pseudomonas putida* involves the metW, metX, metZ, metH and metE gene products. *Arch Microbiol* 2001;176:151–4. 10.1007/s002030100293 11479715

[77] Peck HD Jr . Enzymatic basis for assimilatory and dissimilatory sulfate reduction. *J Bacteriol* 1961;82:933–9. 10.1128/jb.82.6.933-939.1961 14484818 PMC279279

[78] Hao Y, Pan X, You J et al. Microbial production of branched chain amino acids: advances and perspectives. *Bioresour Technol* 2024;397:130502. 10.1016/j.biortech.2024.130502 38417463

[79] Bull CT, Shetty KG, Subbarao KV. Interactions between myxobacteria, plant pathogenic fungi, and biocontrol agents. *Plant Dis* 2002;86:889–96. 10.1094/PDIS.2002.86.8.889 30818644

[80] Contreras-Moreno FJ, Munoz-Dorado J, Garcia-Tomsig NI et al. Copper and melanin play a role in *myxococcus xanthus* predation on *Sinorhizobium meliloti*. *Front Microbiol* 2020;11:94. 10.3389/fmicb.2020.00094 32117124 PMC7010606

[81] Kroos L, Wall D, Islam ST et al. Milestones in the development of *myxococcus xanthus* as a model multicellular bacterium. *J Bacteriol* 2025;207:e0007125. 10.1128/jb.00071-25 40525847 PMC12288465

[82] Scheel M, Zervas A, Rijkers R et al. Abrupt permafrost thaw triggers activity of copiotrophs and microbiome predators. *FEMS Microbiol Ecol* 2023;99:fiad123. 10.1093/femsec/fiad123 PMC10599396 37796894

[83] Le PT, Pontarotti P, Raoult D. *Alphaproteobacteria* species as a source and target of lateral sequence transfers. *Trends Microbiol* 2014;22:147–56. 10.1016/j.tim.2013.12.006 24461455

[84] Msaddak A, Duran D, Rejili M et al. Diverse bacteria affiliated with the genera *microvirga, Phyllobacterium*, and *Bradyrhizobium* Nodulate *Lupinus micranthus* growing in soils of northern Tunisia. *Appl Environ Microbiol* 2017;83:e02820-16. 10.1128/AEM.02820-16 PMC5335532 28062461

[85] Akbar S, Phillips KE, Misra SK et al. Differential response to prey quorum signals indicates predatory specialization of myxobacteria and ability to predate *Pseudomonas aeruginosa*. *Environ Microbiol* 2022;24:1263–78. 10.1111/1462-2920.15812 34674390 PMC9257966

[86] Lloyd DG, Whitworth DE. The myxobacterium *myxococcus xanthus* can sense and respond to the quorum signals secreted by potential prey organisms. *Front Microbiol* 2017;8:439. 10.3389/fmicb.2017.00439 28352265 PMC5348527

[87] Khanal , Pokharel S, Shehata N, Ahearne A et al. Establishing conserved biosynthetic gene clusters of the phylum *Myxococcota*. *Appl Environ Microbiol* 2025;**92**:e02151-25. 10.1128/aem.02151-25:e0215125 PMC12766979 41378891

[88] Lindsay PL, Ivanov S, Pumplin N et al. Distinct ankyrin repeat subdomains control VAPYRIN locations and intracellular accommodation functions during arbuscular mycorrhizal symbiosis. *Nat Commun* 2022;13:5228. 10.1038/s41467-022-32124-3 36064777 PMC9445082

[89] Kumagai H, Hakoyama T, Umehara Y et al. A novel ankyrin-repeat membrane protein, IGN1, is required for persistence of nitrogen-fixing symbiosis in root nodules of *Lotus japonicus*. *Plant Physiol* 2007;143:1293–305. 10.1104/pp.106.095356 17277093 PMC1820915

[90] Hamilton W, Massey J, Hardy E et al. *Wolbachia* uses ankyrin repeats to target specific fly proteins. *mBio* 2026;**17**:e00172-26. 10.1128/mbio.00172-26 PMC13170367 42003611

[91] Siozios S, Ioannidis P, Klasson L et al. The diversity and evolution of *Wolbachia* ankyrin repeat domain genes. *PLoS One* 2013;8:e55390. 10.1371/journal.pone.0055390 23390535 PMC3563639

[92] Iturbe-Ormaetxe I, Burke GR, Riegler M et al. Distribution, expression, and motif variability of ankyrin domain genes in *Wolbachia pipientis*. *J Bacteriol* 2005;187:5136–45. 10.1128/JB.187.15.5136-5145.2005 16030207 PMC1196006

[93] Pan X, Lührmann A, Satoh A et al. Ankyrin repeat proteins comprise a diverse family of bacterial type IV effectors. *Science* 2008;320:1651–4. 18566289 10.1126/science.1158160 PMC2514061

[94] Simeon R, Jiang M, Chamoun-Emanuelli AM et al. Selection and characterization of ultrahigh potency designed ankyrin repeat protein inhibitors of *C. Difficile* toxin B. *PLoS Biol* 2019;17:e3000311. 31233493 10.1371/journal.pbio.3000311 PMC6590788

[95] Rouhizohrab N, Mohammadipanah F. Suppression of predominant interfering bacteria in the purification process of myxobacteria. *Iran J Microbiol* 2022;14:721–9. 10.18502/ijm.v14i5.10968 36531805 PMC9723438

[96] Brock DA, Douglas TE, Queller DC et al. Primitive agriculture in a social amoeba. *Nature* 2011;469:393–6. 10.1038/nature09668 21248849

[97] Brock DA, Read S, Bozhchenko A et al. Social amoeba farmers carry defensive symbionts to protect and privatize their crops. *Nat Commun* 2013;4:2385. 10.1038/ncomms3385 24029835

[98] DiSalvo S, Haselkorn TS, Bashir U et al. *Burkholderia* bacteria infectiously induce the proto-farming symbiosis of *Dictyostelium* amoebae and food bacteria. *Proc Natl Acad Sci USA* 2015;112:E5029–37. 10.1073/pnas.1511878112 26305954 PMC4568666

[99] Felsenstein J . Confidence limits on phylogenies: an approach using the bootstrap. *Evolution* 1985;39:783–91. 10.1111/j.1558-5646.1985.tb00420.x 28561359

[100] Stecher G, Tamura K, Kumar S. Molecular evolutionary genetics analysis (MEGA) for macOS. *Mol Biol Evol* 2020;37:1237–9. 10.1093/molbev/msz312 31904846 PMC7086165

