# Supplementary material for: Predator avoidance promotes interbacterial symbiosis with myxobacteria in polymicrobial communities
Source: ISME J. 2026 Jun 1;20(1):wrag140. doi: 10.1093/ismejo/wrag140 (PMC13293253; doi:10.1093/ismejo/wrag140)
Supplement: Supplementary_material_wrag140 [file supplementary_material_wrag140.zip › consortia_ISME_SI_rev1.docx]

**Predator avoidance promotes inter-bacterial symbiosis with myxobacteria in polymicrobial communities**

Shailaja Khanal Pokharel*, Sheila Walsh***,** Nawal Shehata, Andrew Ahearne, Daniel Belin, Britney Larson, Benjamin Tabor, Daniel Wall, and Cole Stevens

**Supplemental Data**

**Supplemental Table S1:** Assembly statistics for WIMLSP1

| **Scaffold ID (IMG/MER)** | **Sequence length (bp)** | **GC content** | **Gene count** |
| --- | --- | --- | --- |
| Ga0669685_01 | 12,981,101 | 0.69 | 10,693 |
| Ga0669685_02 | 2,513 | 0.64 | 6 |
| Ga0669685_03 | 3,923,152 | 0.61 | 3,882 |
| Ga0669685_04 | 2,538 | 0.68 | 2 |
| Ga0669685_05 | 2,575 | 0.70 | 3 |
| Ga0669685_06 | 2,776 | 0.69 | 2 |

**Supplemental Table S2:** Assembly statistics for WIMLSP2

| **Scaffold ID (IMG/MER)** | **Sequence length (bp)** | **GC content** | **Gene count** |
| --- | --- | --- | --- |
| Ga0646599_01 | 12,928,281 | 0.69 | 10,576 |
| Ga0646599_02 | 4,285,839 | 0.62 | 4,146 |
| Ga0646599_03 | 5,931 | 0.56 | 13 |

**Supplemental Table S3:** Assembly statistics for FLWO

| **Scaffold ID (IMG/MER)** | **Sequence length (bp)** | **GC content** | **Gene count** |
| --- | --- | --- | --- |
| Ga0654980_01 | 13,960,269 | 0.68 | 11,322 |
| Ga0654980_02 | 2,321 | 0.65 | 2 |
| Ga0654980_03 | 2,877 | 0.69 | 2 |
| Ga0654980_04 | 1,081 | 0.66 | 3 |
| Ga0654980_05 | 1,603 | 0.63 | 2 |
| Ga0654980_06 | 1,183 | 0.64 | 1 |
| Ga0654980_07 | 3,818,472 | 0.61 | 3,738 |
| Ga0654980_08 | 1,347 | 0.67 | 1 |
| Ga0654980_09 | 5,895 | 0.56 | 14 |

**Supplemental Table S4:** Assembly statistics for DLMAZ

| **Scaffold ID (IMG/MER)** | **Sequence length (bp)** | **GC content** | **Gene count** |
| --- | --- | --- | --- |
| Ga0646598_01 | 44,387 | 0.58 | 50 |
| Ga0646598_02 | 12,010,403 | 0.69 | 10,262 |
| Ga0646598_03 | 19,741 | 0.71 | 23 |
| Ga0646598_04 | 158,443 | 0.68 | 86 |
| Ga0646598_05 | 4,194 | 0.68 | 7 |
| Ga0646598_06 | 12,788 | 0.72 | 13 |
| Ga0646598_07 | 133,616 | 0.59 | 113 |
| Ga0646598_08 | 7,740 | 0.72 | 12 |
| Ga0646598_09 | 6,295 | 0.70 | 9 |
| Ga0646598_10 | 4,131,692 | 0.61 | 4,039 |

**Supplemental Table S5:** Swarm consortia details from assembled data

| **Swarm consortia** | **contigs** | **# of bases** | **# of RNA genes** | **# of protein coding sequences** | **Estimated # of genomes** |
| --- | --- | --- | --- | --- | --- |
| WIMLSP1 | 6 | 16,914,655 | 182 | 14,387 | 2 |
| WIMLSP2 | 3 | 17,220,051 | 181 | 14,534 | 2 |
| FLWO | 9 | 17,795,048 | 180 | 14,889 | 2 |
| DLMAZ | 10 | 16,529,299 | 156 | 14,442 | 2 |

**Supplemental Table S6:** dDDH data for swarm consortia myxobacteria. dDDH (d_4_, in %) values provided by TYGS analysis of corresponding MAGs. dDDH values were not calculated for cells with “n.d.”

|  | **A. WIMLSP1** | **A. WIMLSP2** | **A.FLWO** | **C.DLMAZ** | **A.gephyra** | **A.lansingense** | **C.fuscus** |
| --- | --- | --- | --- | --- | --- | --- | --- |
| **A.WIMLSP1** | 100 | 92.7 | 34.8 | 25.3 | 45.9 | 36.2 | 24.9 |
| **A.WIMLSP2** | 92.7 | 100 | 34.8 | 25.1 | 45.7 | 36.2 | 24.9 |
| **A.FLWO** | 34.8 | 34.8 | 100 | 25.2 | 35.4 | 44.9 | 24.9 |
| **C.DLMAZ** | 25.3 | 25.1 | 25.2 | 100 | 25.2 | 25.9 | 52.3 |
| **A.gephyra** | 45.9 | 45.7 | 35.4 | 25.2 | 100 | n.d. | n.d. |
| **A.lansingense** | 36.2 | 36.1 | 44.9 | 25.9 | n.d. | 100 | n.d. |
| **C.fuscus** | 24.9 | 24.9 | 24.9 | 52.3 | n.d. | n.d. | 100 |

**Supplemental Table S7:** dDDH data for swarm consortia *Microvirga*. dDDH (d_4_, in %) values provided by TYGS analysis of corresponding MAGs. dDDH values were not calculated for cells with “n.d.”

|  | **M. WIMLSP1** | **M. WIMLSP2** | **M.FLWO** | **M.DLMAZ** | **M.guangxiensis** | **M.solisilvae** | **M.vignae** |
| --- | --- | --- | --- | --- | --- | --- | --- |
| **M.WIMLSP1** | 100 | 25.9 | 38 | 25.9 | 30.3 | 28.5 | 27.8 |
| **M.WIMLSP2** | 25.9 | 100 | 25.6 | 38.5 | 24.9 | 25.3 | 24.8 |
| **M.FLWO** | 38 | 25.6 | 100 | 25.8 | 30.2 | 28.5 | 27.7 |
| **M.DLMAZ** | 25.9 | 38.5 | 25.8 | 100 | 25.2 | 25.5 | 24.9 |
| **M.guangxiensis** | 30.3 | 24.9 | 30.2 | 25.2 | 100 | n.d. | n.d. |
| **M.solisilvae** | 28.5 | 25.3 | 28.5 | 25.5 | n.d. | 100 | n.d. |
| **M.vignae** | 27.8 | 24.8 | 27.7 | 24.9 | n.d. | n.d. | 100 |

**Supplemental Table S8:** Genes that are phylogenetically associated with Myxococcota that are present in *Microvirga* MAGs.

| **MAG** | **AA sequence** | **annotation** | **Top BLASTP hit (species** | **2nd BLASTP hit (species)** |
| --- | --- | --- | --- | --- |
| **M.WIMLSP1** | MLKLAFAAAVGTTLLFASGAQALETQNGLAFNGLAFNGLAFNGLAFNGLAFNGAAADGVAGELRSAPALQATTVILKDGERVSLK | hypothetical protein | Microvirga sp. ACRRW | Pyxidicoccus sp. 3LG |
|  | MLMNQDEYDKHLKGFMITGCTVRSKDVFYLVAITDSPNRARPESDLTTRVIPYFFEKIEKRWGHINYHGYSRTLAGASLYPESKFVGVDRGGQVMVVGGGKMEIEDIAGGRTGPIRGSVNRVRTINGFIHVCSNNRGLARRDGTDRWTSLCADLPVKPNPNGFGEVYGFNDFDAFDNGEFYCVGGQSDVWRFDGANWTPIDVPGDHNGASNFLTKAGSKIARVPLHSVCCAGDGYLYIGGPDGGVWKGRNEQWKLIHDDRLSLPFKDIVWFQDRVYCTSRYGLWEIVNDEVRPCDVPEEISICSGNLAVADGIMLLAGECGAAYHDGREWKLIFNTSSFT | WD40/YVTN/BNR-like beta propeller repeat protein | Microvirga sp. ACRRW | Archangium sp. |
|  | MIFRHLRGGLRAVLFTLLGLLGYPSLVSAAGPDMYFDAPADKQMALAVASGDIETMTALLSSKAVDPQAIGRKATSWIEIAIIADQKKAFDTLMKWNALGPPKGKIAGQAMYSATVKGSIRWLERLAAAGASLDNYGGGELLIVTALDTRNEAVLDFYIRNGADLDMPAMAGGSVALSAAMTRRFDMALRFLDLGASPWVMDSLGSTLGSIAERAARVPAWDHSSRMNQHRLELLQRLHAIGFPDPAPTADEGHALRQKKQWPPKAAIKQ | ANKYR superfamily | Microvirga sp. ACRRW | Archangium sp. |
|  | MFKIPEPMLTYWASKAYKKASIADLAEVERLFGTALPASYVEFVTTIGFVVFDDVPGFKIHEYFDYKVGSPEGTEIAQGNIAFLKEPAHIIKAHKILTNRQALEEEEEEDEDFPKFPKNYLPIANDAGQGQILMEFGEHPGRIWYWQENDWAWGLEDNTWLGFVAENFEDFINGLKP | SM1/KNR4 family protein | Archangium violaceum | Candidatus Methylumidphilus sp. |
|  |  |  |  |  |
| **M. WIMLSP2** | MSRTMKYTILVSFVGLLSAGAQAAEIANGSDLNGANLNGANLNGSDLNGSDLNGANLNGASSGRVFLGAQVSALIAPDGTLVTLD | pentapeptide repeat protein | Microvirga sp. | Archangium violaceum |
|  | MMTTIDDIRRRDGFLTIEETLSLCDRNTIYDPYSTLISRHARIGSGNILYPCTTIRCSQDSSCEVGDRNIFHSLTMIDACGGAISIGSGNTFGDGGFTAKADRPGAKITIGDRGRYASGASVYGVSHLGTGSQILGQISVIDCVLADGEDFTHADPDERAAVLKGHGAARKLRIGVGEVIFGKSSFDQAGIQRQTDFHPKS | IpxD-like protein | Pyxidicoccus sp. 3LG | Stappiacea bacterium |
|  | MSYPFYLLYGAYLASGLGDWILRIAVPLLIYQVTDSALAMAGAYAVNYLPYLIVTPFGGVLADRVDRRRMLLVGDFFAAGLVVAIILANASGAAALLLYPLLFVLASTAAVYHPGFQSFIPSVVPPDKLARANSLFAAADNGLSLLGPAAAGGIVALLGPVQALYADALSFALSGLLILCIPSAMSAKAQVERKLQGILHDLREGFVYVWHNRILRAGAFLFFFVNFSYDIFYANFIFLLVGIFGLTAVDAGTVISMTGVGALVGSLVAPKLMSRVSSGRLIVACTATAGGLILLLLFVDGALAVGMLWGGVCATQAVIRVAYFTLRQKIVPSNLLGRSVAVTRMISYAAVPLAALSGGWIVQQTGEIRMIVIISGSVMLLSALIAWFTSLGRTPQPALQSSLAT | MFS transporter | Microvirga sp. | Polyangium aurulentum |
|  | MILRQLRGGLRALLFMLLGLFGYPASASAAGPDMYFDSPADKQMALAVASGDIETMTALLSSKAVDPLAIGRKVTSWIEIAVIADQRAAFDALVKWGALGPAKGKIAGQAMYSATIKGSIRWLERLTAAGASLDNHGGGDLLIVQALDTRNEAVLDFYIRNGADLNMPAMAGGSVALSAAMTRRFDMVLKFLDLGASPWVMDSLGSTLGSIAERAARVPAWDHSSRMNQHRLELLRRLHAIGFPDPAPTANEGHALRQKKQWPPKAAIKQ | ANKYR superfamily | Microvirga sp. ACRRW | Archangium sp. |
|  |  |  |  |  |
| **M. FLWO** | MRHRISIGLACILCLMGAGCLDEREKAARTLVKTRACPDCDLTEIKLEAAQLQGAQLAGARLEKADMRKADLREADFSGAILFDTDLRGADLRGAAFREASMTGAQMQGANLEGVDLSGTTLNAIDLSGVNFQGANLRGAKLSEARLNGINGPYRRDPPAHFYAVPAGGADLRGADLSGADLSGAYLSKADLRDAKLAGANLRDAHLDQADLRGADINGADLKGAILDHATWIDGSICAEKSIGRCRRP | pentapeptide repeat protein | Corallococcus sp. | Corallococcus silvisoli |
|  | MKASKWQLKQRWHEEAGALVLKRVQDILLADDTRKLPLGIPDILSGLPFRDEVASGRDFRGIELEGGLTSLDLSGCDFSYAKLTLNFIRCDLSEANFEEATLGGIIFDKATRANFRRAKMRHCSLVGLNAQDCCFDEAILSNASFEKACLQGSKFRNANCKGASFVSANLLGCDFQGANLNECPFQGVILDRSTNLRGASLVGLFYHEHRSIDGKLVLPKTDWRLATHDETTRTEA | pentapeptide repeat protein | Hyalangium gracile | Archangium sp. |
|  | MLKTAFAATVGTTLLFGSGAQALETQNGLAFNGLAFNGLAFNGLAFNGLAFNGAATDGVAEELRSAPALQATTVILKDGEHVSLK | hypothetical protein | Microvirga sp. ACRRW | Pyxidicoccus sp. 3LG |
|  |  |  |  |  |
| **M. DLMAZ** | MACAIGLYHRVKARIPTIAVICTDANSCYRLAFARHGVAEAHVQSKAHTH | transposase | Corallococcus sp. AB045 | Alphaproteobacteria bacterium |
|  | MTCHHCGSTAFRKNGHCAGVQRYVCHACHRSFSANGERFSKAVKAQALDM | transposase | Corallococcus sp. AB045 | Accumulibacter sp. |
|  | MGVLERLVLTDSQWARIAPLIIGRPDQKGSTGRDNRMFVEGVLWIVRTGS | transposase | Microvirga sesbaniae | Corallococcus sp. AB045 |
|  | MACHHCGSSAFCKNGHTRGVQRYRCHACHRSFSANGERFSKTVKAQALDMYLNNVGLRKIARFTGASPPAVLKWIKKAATALAAQLEQAKAQVHDELPDVIEMDEIYTFVQKNSSAPSYGLLILDGRAVLLRTSSATGA | transposase | Corallococcus sp. AB045 | Accumulibacter sp. |
|  | MSSAIGLYRRVKQAVPAVALICTDANSCYRLAFERYRVPEAHVQSKAHTH | transposase | Corallococcus sp. AB045 | Mesorhizobium sp. 8 |
|  | MLLDQTAYDAYFKGFILIDCVIRSKDIFYFVLVSDLKRTRSESDRKTRIVAHFLKSSDKPWRRADYEGFAKVFAGASQLPASKFVGVDRGAQVMLIGSGSLENEDIPAGKQGPIRGAIRKIKTINGYAHVCSGYRGFARRDGPNLWTSLVKNLNFMPDPDKDSGIYGFADFDAFNDRDFYCVGGHSDAWHFDGETWTQLDFPGDPSQIPESLIDPSTPGVPLEAVCCAGNGYVYIGGPGGTVWKGRKNSWTLIHRDSMSLPLRDMVWFKDRVYCTSDYGLWEIVDDQLRPCDIPDEIRVCSGHLSVCDGVMLLAGIYGAAYHDGNRWHLIFDTGQF | WD40/YVTN/BNR-like beta propeller repeat protein | Microvirga sp. ACRRW | Archangium sp. |

**Table S9:** Annotated LuxI homologs from *Archangium* MAGs that share homology with *Microvirga*. *Excluding LuxI homologous from Myxococcota.

| **feature annotation** | **MAG source** | **most homologous protein***  **(BLASTP)** | **amino acid**  **% identity**  **(BLASTP)** | **LuxI**  **% identity between consortia members**  **(BLASTP)** |
| --- | --- | --- | --- | --- |
| AHL synthase | *Archangium* WIMLSP1 | WP_441998110.1  (*Microvirga* sp. 2TAF3) | 73.24 | 43.55 |
| AHL synthase | *Archangium*  WIMLSP2 | WP_441998110.1  (*Microvirga* sp. 2TAF3) | 73.24 | 45.76 |

**Table S10:** Features from *Microvirga* MAGs that share homology with *Archangium*. Features with high homology present in both members of a swarm consortia are bolded.

| **feature annotation** | **MAG source** | **homologous *Archangium* protein** | **amino acid**  **% identity**  **(BLASTP)** | **presence in cognate consortia**  ***Archangium*** | **nucleotide**  **% identity between consortia members**  **(BLASTN)** |
| --- | --- | --- | --- | --- | --- |
| WD40/YVTN/BNR-like beta propeller repeat protein | *Microvirga* WIMLSP1 | WP_329727439.1  (*Archangium* sp.) | 52.62 | not present | n/a |
| **ankyrin repeat domain-containing protein** | ***Microvirga* WIMLSP1** | **WP_375769195.1**  **(*Archangium gephyra)*** | **64.58** | **present**  **(60.08% identity; 298 max score)** | **66.67**  **(82% coverage)** |
| SMI1/KNR4 family protein | *Microvirga*  WIMLSP1 | WP_203409848.1  (*A. violaceum*) | 61.36 | present  (25.3% identity; 41.6 max score) | n.d. |
| **ankyrin repeat domain-containing protein** | ***Microvirga* WIMLSP2** | **WP_375769195.1**  **(*A. gephyra)*** | **64.17** | **present**  **(60.47% identity; 301 max score)** | **68.94**  **(67% coverage)** |
| pentapeptide repeat-containing protein | *Microvirga* FLWO | WP_324950690.1  (*Archangium* sp.) | 50.43 | present  (32.82% identity; 59.3 max score) | 80.25  (31% coverage) |
| WD40/YVTN/BNR-like beta propeller repeat protein | *Microvirga* DLMAZ | WP_329727439.1  (*Archangium* sp.) | 56.3 | not present | n/a |

**Table S11:** Auxotrophies of consortia *Archangium* (bolded) and monoculture *Archangium*. *L-isoleucine auxotrophy in *Archangium* was predicted by GapMind analysis but were not observed in metabolic models(50).

|  | **L-His** | **L-Leu** | **L-Val** | **L-Ile^*^** | **folate** | **riboflavin** |
| --- | --- | --- | --- | --- | --- | --- |
| ***Archangium* sp. WIMLSP1** | **A** | **A** | **A** | **A** | **A** | **A** |
| ***Archangium* sp. WIMLSP2** | **A** | **A** | **A** | **A** | **A** | **A** |
| ***Archangium* sp. FLWO** | **A** | **A** | **A** | **A** | **A** | **A** |
| *Archangium* sp. PVMSAZ | **A** | **A** | **A** | **A** | **A** | **A** |
| *Archangium* sp. SCPoplar1 | **A** | **A** | **A** | **A** | **A** | **A** |
| *Archangium lansingense* | P | **A** | **A** | **A** | P | P |
| *Archangium gephyra* | P | **A** | **A** | **A** | P | P |
| ***Cystobacter* sp. DLMAZ** | **A** | P | P | P | P | P |
| *Cystobacter ferrugineus* | P | P | P | P | P | P |

**
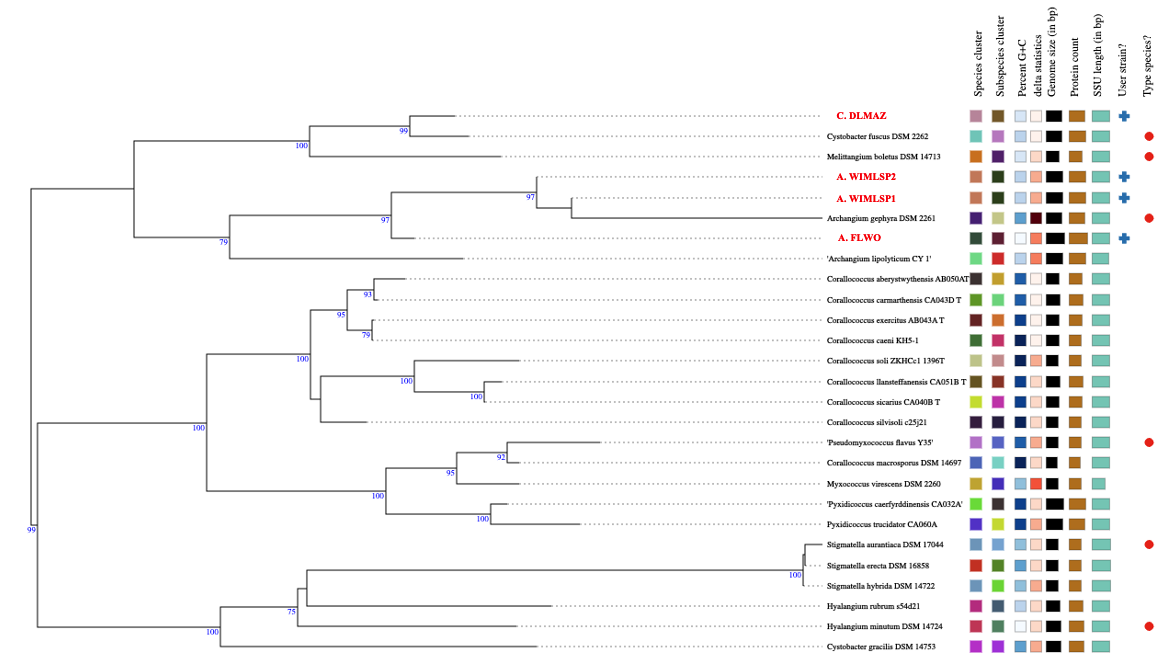
**

**Supplemental Figure S1:** Genome BLAST Distance Phylogeny (GBDP) tree generated from myxobacterial 16S rDNA gene sequences at the Type Strain Genome Server (TYGS).

**
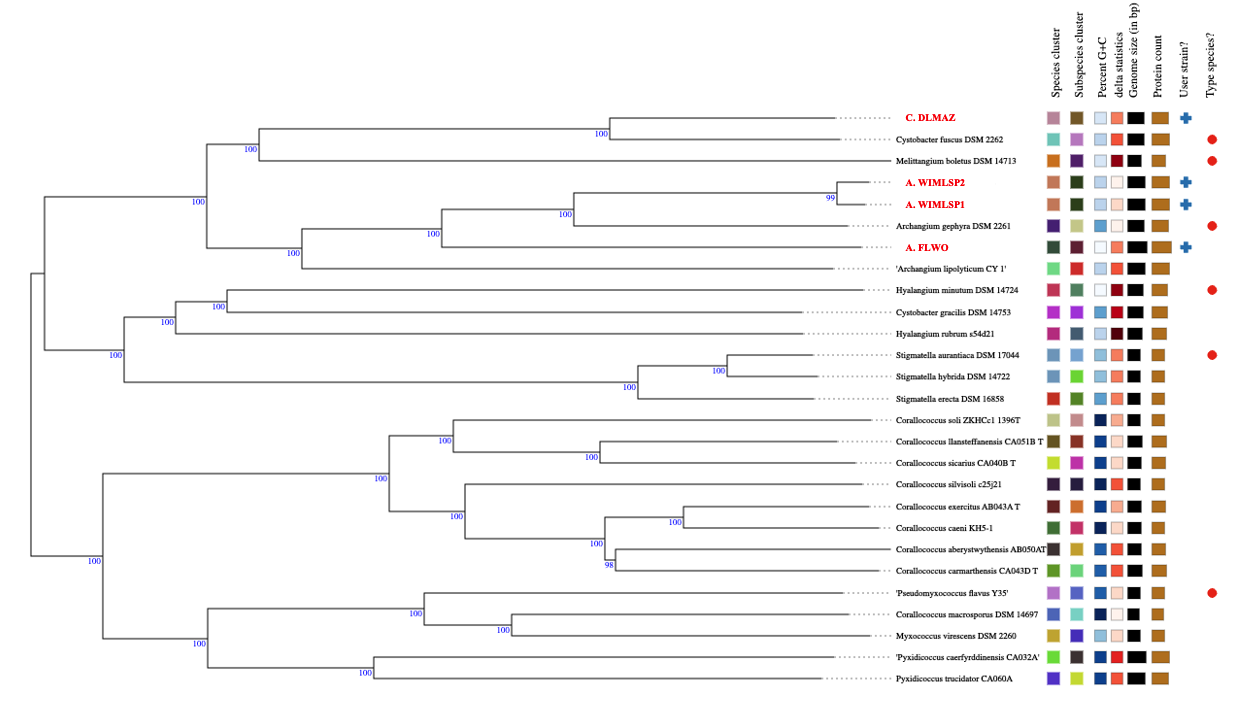
**

**Supplemental Figure S2:** Genome BLAST Distance Phylogeny (GBDP) tree generated from myxobacterial MAG sequences at the Type Strain Genome Server (TYGS).

**
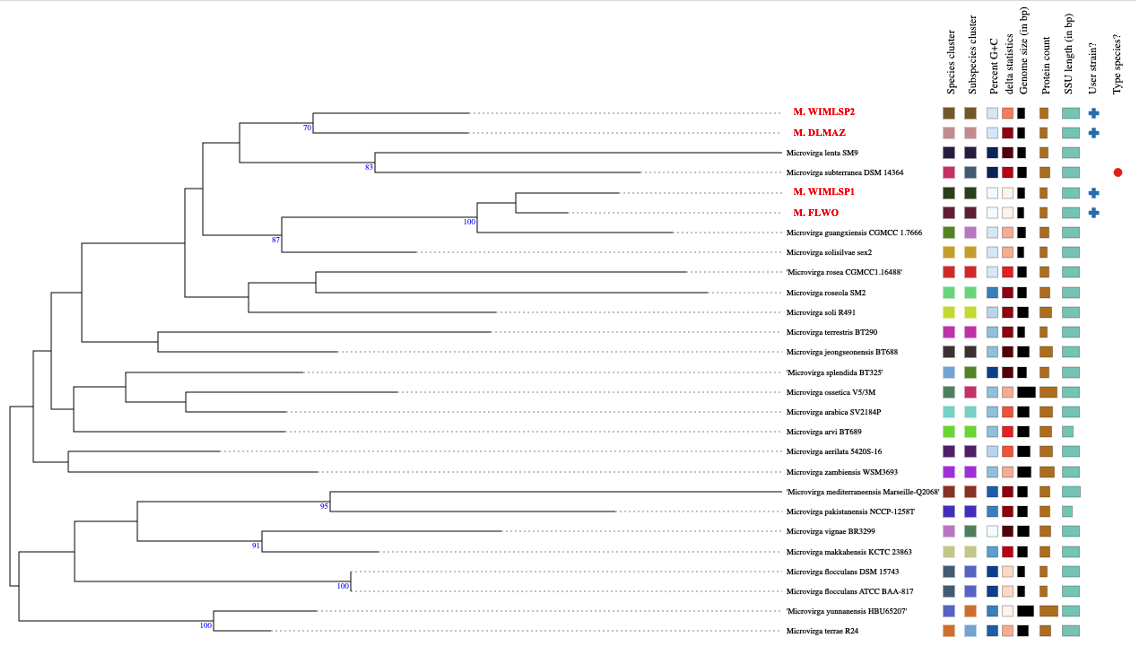
**

**Supplemental Figure S3:** Genome BLAST Distance Phylogeny (GBDP) tree generated from *Microvirga* 16S rDNA gene sequences at the Type Strain Genome Server (TYGS).

**
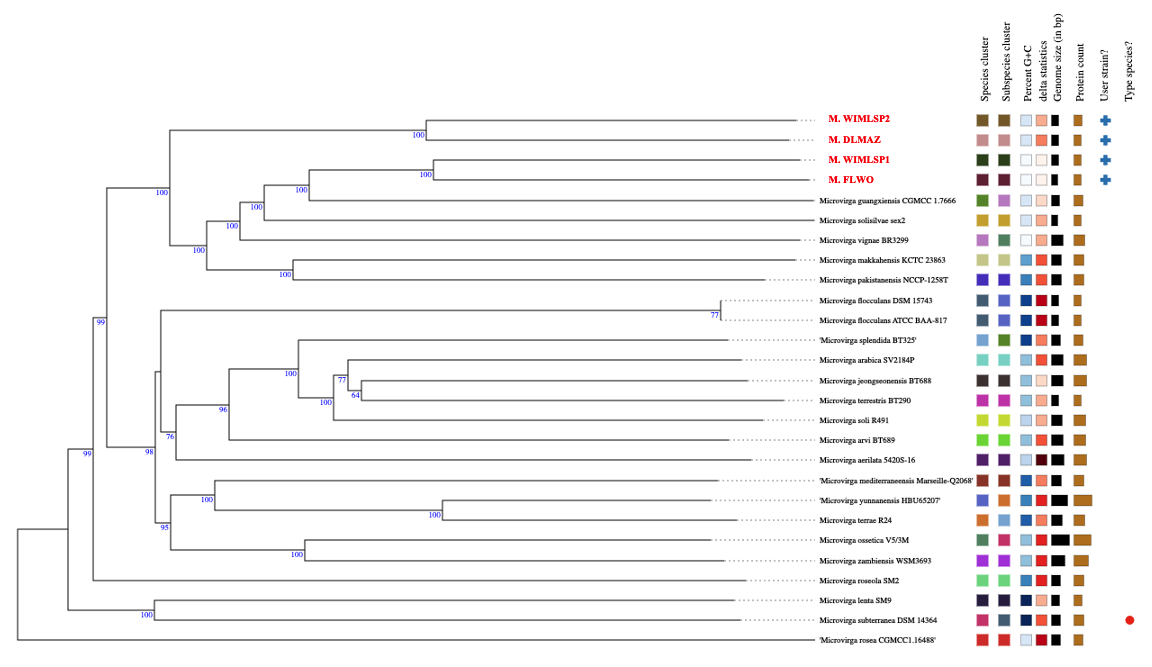
**

**Supplemental Figure S4:** Genome BLAST Distance Phylogeny (GBDP) tree generated from *Microvirga* MAG sequences at the Type Strain Genome Server (TYGS).

**
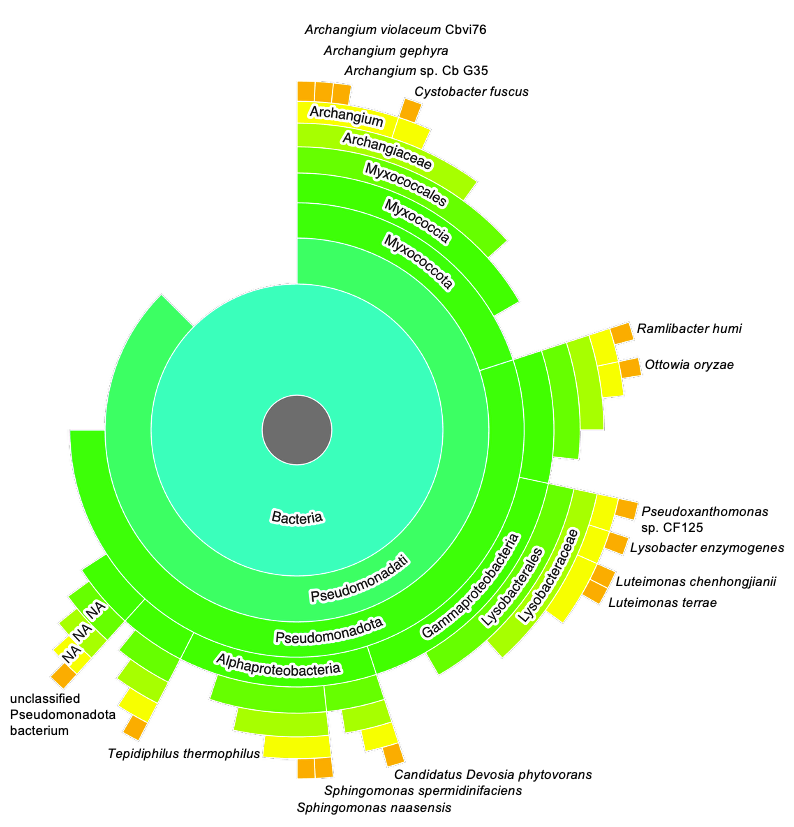
**

**Supplemental Figure S5:** Phylogenetic distribution of ANKYR proteins identified with EFI-EST analysis.

**
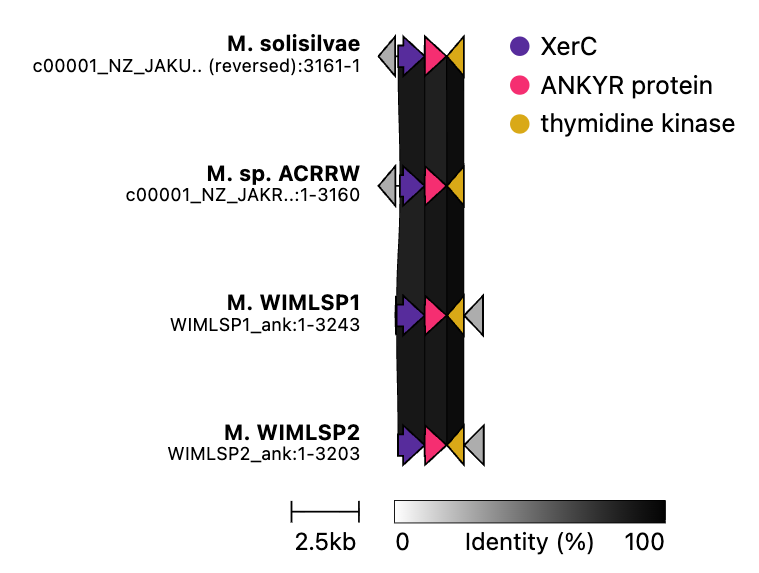
**

**Supplemental Figure S6:** Conserved spatial organization of XerC and ANKYR proteins in genomes of *M.* WIMLSP1, *M.* WIMLSP2, *M. solisilvae*, and *M.* sp. ACRRW. Figure rendered with clinker.

**
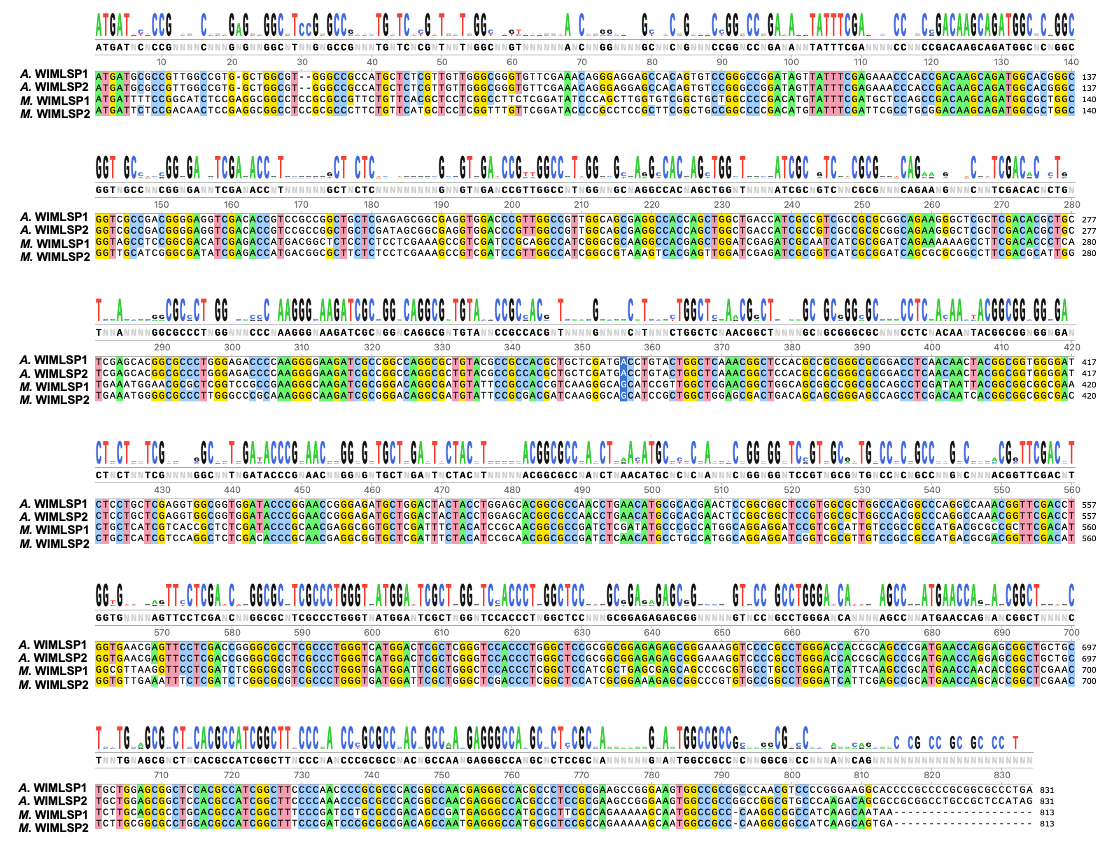
**

**Supplemental Figure S7:** Alignment of genes encoding ANKYR proteins from WIMLSP1 and WIMLSP2 generated with MEGA X using Clustal.

**
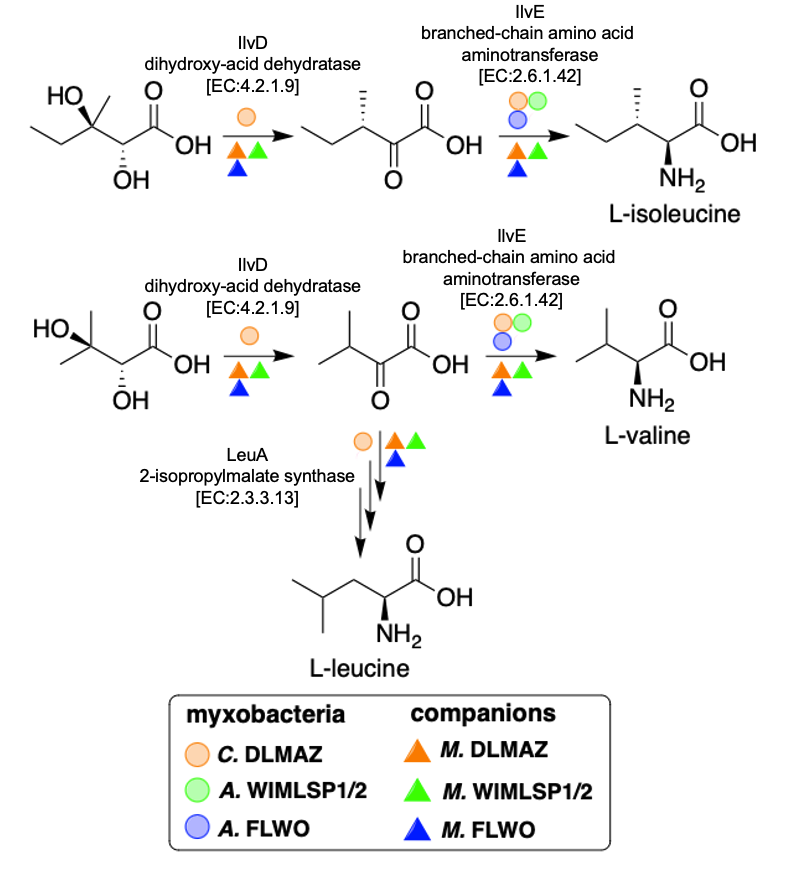
Supplemental Figure S8:** BCAA biosynthetic pathways from swarm consortia depicting BCAA auxotrophy in *Archangium*.
